# Voltage-gated sodium channels in cancers

**DOI:** 10.1186/s40364-024-00620-x

**Published:** 2024-07-25

**Authors:** Hengrui Liu, Jieling Weng, Christopher L.-H. Huang, Antony P. Jackson

**Affiliations:** 1 Department of Biochemistry, Hopkins Building, University of Cambridge, Tennis Court Road, Cambridge, CB2 1QW UK; 2 Department of Pathology, The Second Affiliated Hospital of Guangzhou Medical University, Guangzhou, China; 3 Physiological Laboratory, University of Cambridge, Downing Street, Cambridge, CB2 3EG UK

**Keywords:** VGSC, Cancer, TCGA

## Abstract

Voltage-gated sodium channels (VGSCs) initiate action potentials in electrically excitable cells and tissues. Surprisingly, some VGSC genes are aberrantly expressed in a variety of cancers, derived from “non-excitable” tissues that do not generate classic action potentials, showing potential as a promising pharmacological target for cancer. Most of the previous review articles on this topic are limited in scope, and largely unable to provide researchers with a comprehensive understanding of the role of VGSC in cancers. Here, we review the expression patterns of all nine VGSC α-subunit genes (SCN1A-11A) and their four regulatory β-subunit genes (SCN1B-4B). We reviewed data from the Cancer Genome Atlas (TCGA) database, complemented by an extensive search of the published papers. We summarized and reviewed previous independent studies and analyzed the VGSC genes in the TCGA database regarding the potential impact of VGSC on cancers. A comparison between evidence gathered from independent studies and data review was performed to scrutinize potential biases in prior research and provide insights into future research directions. The review supports the view that VGSCs play an important role in diagnostics as well as therapeutics of some cancer types, such as breast, colon, prostate, and lung cancer. This paper provides an overview of the current knowledge on voltage-gated sodium channels in cancer, as well as potential avenues for further research. While further research is required to fully understand the role of VGSCs in cancer, the potential of VGSCs for clinical diagnosis and treatment is promising.

**Supplementary Information:**

The online version contains supplementary material available at 10.1186/s40364-024-00620-x.

## Voltage-gated sodium channels

In recent years, ion channels have emerged as a promising new target for cancer management [1–14]. Many cancer cells and tissues possess a wide range of ion channels and these may be involved in various stages of cancer development, progression, and response to treatment. The membrane potential of cancer and non-cancer cells differ [15, 16]. It has been reported that membrane depolarization facilitates cell proliferation, through actions on initiation of mitosis and DNA synthesis [17, 18]. Interestingly, some tumor tissues have a higher concentration level of sodium ions than their normal tissues, whereas their potassium ion concentrations were similar [10, 19–21]. This suggests that intracellular sodium ions may be partially determining the abnormal membrane potentials in cancer cells. Therefore, sodium permeable channels might play a critical role in cancers.

Voltage-gated sodium channels (VGSCs) are transmembrane proteins that increase the permeability of sodium ions across membranes. The permeability of these channels depends on the voltage drop across the membrane. In a typical neuronal action potential, VGSCs remain closed until the membrane potential reaches a threshold, at which point they transiently become permeable to sodium ions. The resulting influx of sodium ions leads to membrane potential depolarization, which regeneratively triggers the opening of more sodium channels, further depolarizing the membrane potential. Within milliseconds, the sodium channels transition to an ion-impermeable inactivated state, while potassium channels are activated. Both events contribute to the restoration of the resting membrane potential [22].

**Fig. 1:**
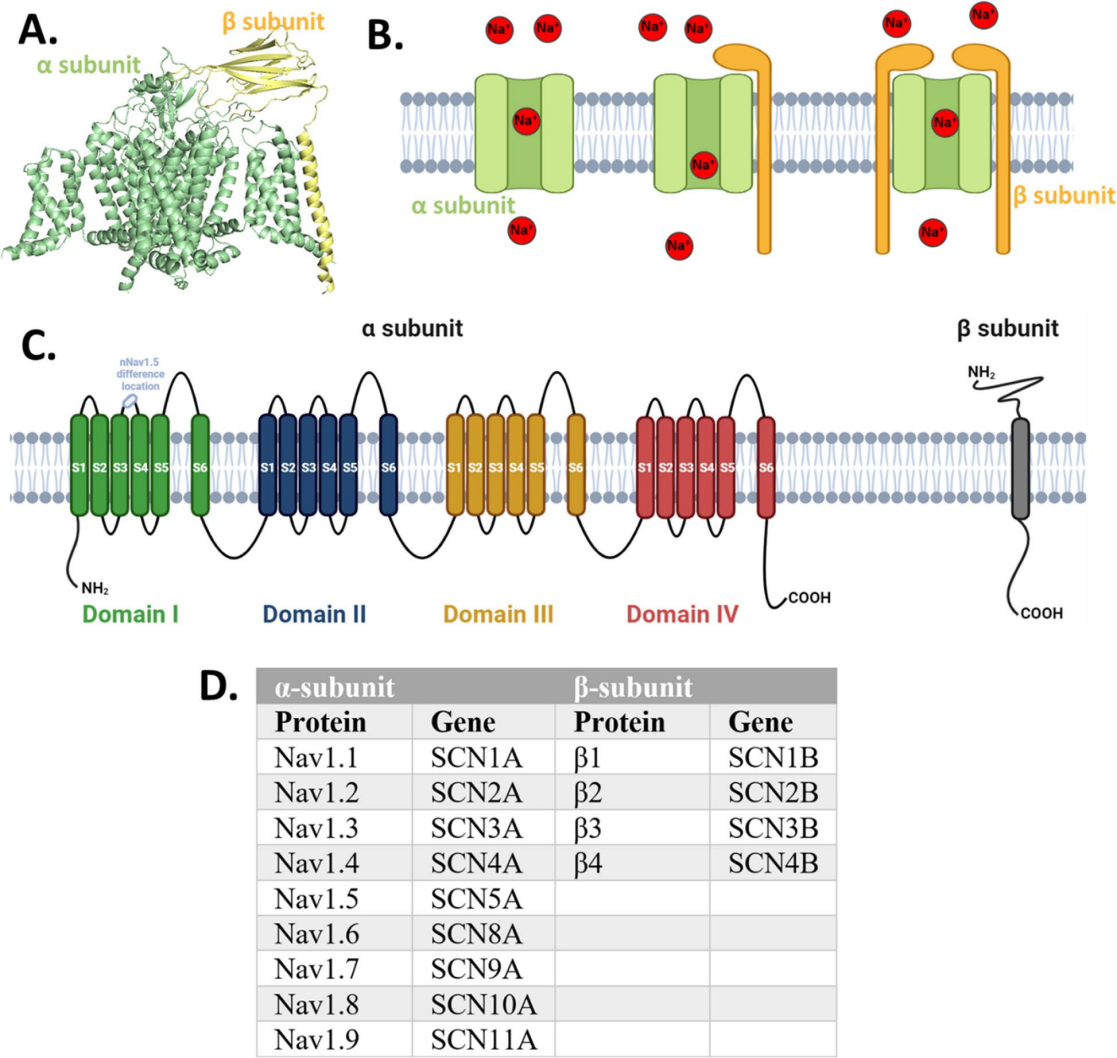
Voltage-gated sodium channels (VGSC). **A** An example structure of the VGSC α-subunit/β-subunit protein complex. **B** Cartoon illustration of VGSC α-subunit and β-subunit proteins within the membrane. **C** Topology diagram of VGSC α-subunit and β-subunit proteins. **D** Protein and gene names of VGSC molecules. Images in Figures **B **and** C** were created using using the BioRender.com

Mammalian Nav channels are formed by a large pseudo-tetrameric pore-forming α-subunit (260 kDa) that can associate with one or more β-subunits (30–40 kDa) (Fig. 1A&B). The α subunit has four homologous domains (DI-IV), each containing six transmembrane helices (S1-6). The S1-4 form the voltage-sensing module, which responds to membrane potential changes, while the S5-6 helices of each of the DI-IV form the pore module (Fig. 1C). To date, a total of nine types of Nav channel α subunit isoforms (Nav1.1, Nav1.2, Nav1.3, Nav1.4, Nav1.5, Nav1.6, Nav1.7, Nav1.8, and Nav1.9) and four types of β subunit isoforms (β1, β2, β3, and β4) have been identified in different human tissues [23, 24] (Fig. 1D). Generally, Nav channel α subunits are divided into two groups, Tetrodotoxin (TTX)-sensitive (Nav1.1, Nav1.2, Nav1.3, Nav1.4, Nav1.6, and Nav1.7) and TTX-resistant (Nav1.5, Nav1.8, and Nav1.9), based on their electrophysiological properties in the presence of the blocker TTX. Neonatal alternative splice variants of the α subunit have been found in some cancer types, such as neonatal Nav1.5 (nNav1.5) which differ from the adult isoform near the S3-S4 linker of Domain I (Fig. 1C). VGSC activation has been suggested to be associated with cancer, leading to an increased interest in this area of research. Despite the potential implications of this connection, the state of research in this field has been disorganized, necessitating an up-to-date summary.

## Overview of previous studies on VGSC in cancers

Following the PRISMA guidelines [25], articles in English in the PubMed database were reviewed up to September 2023 that provided evidence of voltage-gated sodium channels in cancer. Some articles were not reviewed, such as papers studying local anesthetics on cancer cells [26], because they might have additional pharmacological targets [4]. The experimental evidence of VGSC in cancer has been reported since 1995. In the past 10 years, there have been approximately 2–6 research articles published each year in the field except for 2019 which has 11 papers published (S-Fig. 1**)**. It is noteworthy that nearly a quarter of publications on the role of VGSCs in cancer consist of reviews and commentaries [27–47]. This suggests that, while the topic is popular and widely discussed, there are a relatively limited number of experimental studies. Almost all of the previous reviews and commentaries focus on VGSC α-subunits with a few mentioning the β subunits. There are 6 reviews specifically devoted to VGSC in breast cancers [27–32], 3 of which focus on Nav1.5 [28, 29, 32]. There are 6 previous reviews that summarized the effect on cancer migration or invasion [27, 29, 32–36], 4 on drugs [30, 37–39], and 2 on cancer immune [28, 40]. Notably, commentary on the role of VGSC in cancers [18] was published as early as 1986, predating the first research article on the same topic by nine years. This demonstrates that researchers had an interest and were proposing hypotheses regarding the role of VGSC in cancers long before any experimental data was available. Most of the previous review articles on this topic are limited in scope, and largely unable to provide researchers with a comprehensive understanding of the role of VGSC in cancers. A review of the summary of VGSC overexpression mixed in vivo and in vitro cell lines results [37] overlooked the difference between cancer tissues and cancer cell lines. Other papers have summarized in vivo studies only not including the β subunits [33] or proposed hypotheses without a systematic summary [41], and do not provide an in-depth analysis of the effects of VGSC overexpression in both in vivo and in vitro settings. Furthermore, a systematic review of the VGSC inhibitors for cancer [30] was published 8 years ago and an in-time update is now required to summarize VGSC drugs for cancers in papers since then.

## In vitro evidence of VGSCs in cancers

The in vitro studies of VGSCs in cancers collected from the published papers are summarized in Table 1. Breast cancer is the most commonly reported cancer type, followed by prostate cancer, cervical cancer, and colon cancer. Most studies reported the presence of Nav1.5 and Nav1.7 VGSC subtypes, while the β-subunits are rarely reported. Generally, VGSCs were found to promote cancer cell migration and invasion, with a few studies suggesting an effect on cell proliferation.

**Table 1:** In vitro evidence of the impact of VGSCs on cancers

Cancer type	Cell lines	VGSC subtype	Effects of VGSCs	Mechanisms	Reference
Breast	MDA-MB-231 and 4T1	α-subunits	Mediates invasion	/	[48]
Breast	MDA-MB-231 and MCF-7	Nav1.5	Mediates invasion	Decreases sodium currents, epithelial-mesenchymal transition	[49]
Breast	MDA-MB-231	Nav1.5	Mediates migration	Affects Epithelial-mesenchymal transition	[50]
Breast	MDA-MB-231	Nav1.5	Mediates proliferation, migration, and invasion	/	[51]
Breast	MDA-MB-231	Nav1.5	Mediates migration	Decreases Matrix metallopeptidase 9 activity	[52]
Breast	MDA-MB-231	Nav1.5	Mediates invasion	Decreases Matrix metallopeptidase 9 activity	[53]
Breast	MDA-MB-231 and SK-BR-3	Nav1.5	Mediates proliferation and migration	/	[54]
Breast	MDA-MB-231	Nav1.5	Mediates migration	repressor element silencing transcription factor and histone deacetylases	[55]
Breast	MDA-MB-231	Nav1.5	Mediates migration and invasion	Decreases sodium currents	[56]
Breast	MDA-MB-231	α-subunits	Mediates migration	generation of oscillatory intracellular Ca2 + activity	[57]
Breast	MDA-MB-231 and MDA-MB-468	Nav1.5	Mediates adhesion	Sigma-1 receptor activation	[58]
Breast	MCF-7	Nav1.5	Mediates invasion	Decreases sodium currents	[59]
Breast	MDA-MB-231	Nav1.5 and Nav1.7	Mediates proliferation, migration, and invasion	Decreases sodium currents	[60]
Breast	MDA-MB-231	Nav1.5	Mediates migration	Decreases sodium currents, Matrix metalloproteinase type 9 activity, Ki67 activity	[61]
Breast	MDA-MB-231	Nav1.5, Nav1.6, and Nav1.7	Mediates migration	/	[32]
Breast	MDA-MB-231	Nav1.5	Mediates migration and invasion	Decreases sodium currents	[33]
Breast	MDA-MB-231	β1	Mediates migration	Affect the α-subunit via β1 fyn kinase	[34]
Breast	MDA-MB-231 and MCF-7	Nav1.5	Mediates proliferation and invasion	small GTPase RhoA	[35]
Breast	MDA-MB-231	Nav1.5	Mediates migration and invasion	NHE1-dependent H( +) efflux in caveolae	[36]
Breast	MDA-MB-231	Nav1.5	Mediates migration	cAMP-dependent protein kinase A	[37]
Breast	MDA-MB-231	Nav1.5	Mediates invasion	Affect Cysteine Cathepsin signalling	[38]
Breast	MDA-MB-231 and MCF-7	Nav1.5	Mediates migration and invasion	promote the activity of cell invadopodia	[39]
Breast	MDA-MB-231	Nav1.5	Expressed in cancer	/	[40]
Breast	MDA-MB-231	α-subunits	Mediates invasion	/	[41]
Colon	HCT116, HT29, SW480 and SW620	Nav1.5	Mediates invasion	Decreases sodium currents	[45]
Colon	SW480 and DLD1	Nav1.5	Mediates proliferation, migration, and invasion, enhances chemosensitivity	Cell cycle, epithelial-mesenchymal transition, and Ras signaling	[46]
Colon	SW620	Nav1.5	Mediates migration and invasion	ROCK signalling pathway	[47]
Colon	SW620	Nav1.5	Mediates invasion	Hypoxic pathway	[48]
Colon	SW620	Nav1.5	Mediates invasion	Decreases sodium currents	[49]
Ovarian	KURAMOCHI, OVCAR3, OVCAR5, JHOS4, and OVSAHO	α-subunits	Mediates proliferation, enhances chemosensitivity	/	[50]
Ovarian	Caov-3	Nav1.5	Mediates proliferation, migration, and invasion	/	[51]
Ovarian	SKOV3	Nav1.5	Mediates proliferation and migration	/	[51]
Ovarian	Caov-3 and SKOV-3	Nav1.5	Mediates migration and invasion	/	[52]
Endometrial	Primary cancer cell	Nav1.7	Mediates invasion, reduce apoptosis	/	[53]
Prostate	PC3	α-subunits	Mediates proliferation, migration, and invasion	Cell cycle, glucose uptake	[54]
Prostate	PC3M	α-subunits	Mediates migration	generation of oscillatory intracellular Ca2 + activity	[57]
Prostate	PC3 and LNCaP	Nav1.6 and Nav1.7	Expressed in cancer cells	/	[55]
Prostate	LNCaP, C4-2, C4-2B, CWR22Rv-1, DU145, PC-3, and PC-3M	Nav1.1, Nav1.2, Nav1.5, Nav1.6, Nav1.7, Nav1.9	Expressed in cancer cells	/	[56]
Prostate	MAT-LyLu	Nav1.7	Mediates migration, and invasion	/	[57]
Prostate	PC-3 and Mat-LyLu	Nav1.6 and Nav1.7	Mediates proliferation, migration, and invasion	/	[58]
Prostate	PC3M	Nav1.7	Mediates migration	Epidermal growth factor signalling	[59]
Prostate	LNCaP, PC-3, and PC-3M	β1-4	Expressed in cancer	/	[60]
Prostate	RAMA 37, RMP1, RMP1a-lu, RMP2, RMP2c-lu, Du145, and PC3	α-subunits	Mediates migration	/	[63]
Gastric	BGC-823 and MKN-28	Nav1.7	Mediates proliferation and invasion	Regulates extracellular and intracellular pH via increased Na + /H + exchanger-1	[64]
Cervical	SiHa	Nav1.5	Mediates proliferation, migration, and invasion	/	[51]
Cervical	SiHa	β1	Mediates proliferation	Cell cycle	[65]
Cervical	SiHa and CaSki	β1-4	Mediates migration	/	[65]
Cervical	SiHa, CaSki, and HeLa	β4	Mediates invasion	/	[65]
Cervical	C33A, SiHa, CaSki and HeLa	Nav1.6	Mediates invasion	Matrix metalloproteinase type 2 activity	[66]
Cervical	Primary cancer cell	Nav1.2, Nav1.4, Nav1.6, and Nav1.7	/	/	[67]
Lung	H460	Nav1.7	Mediates invasion	epidermal growth factor receptor (EGFR) signalling	[68]
Lung	H23, H460 and Calu-1	Nav1.6 and Nav1.7	Mediates invasion	/	[69]
Lung	H23, H460 and Calu-1	β1 and β3	/	/	[69]
Lung	NCI-H146	α-subunits	Expressed in cancer cells	/	[70]
Oral	SCC-15	Nav1.5	Mediates proliferation, migration, and invasion	Wnt/β-catenin signaling pathway	[73]
Oral	SCC-15 and HSC-3	Nav1.5	Mediates proliferation, migration, and invasion	/	[74]
Liver	HepG2	β3	Mediates proliferation and suppresses apoptosis	Facilitating p53 degradation	[75]
Thyroid	FTC-133 and FTC-238	Nav1.6	Mediates proliferation and invasion	JAK-STAT pathway	[71]
Thyroid	MZ-CRC-1 and TT	Nav1.7	Mediates migration	Decreases sodium currents	[72]

Breast cancer is the most commonly reported cancer type, and numerous studies have demonstrated the expression of Nav1.5 in this type of cancer [32–41, 48–61]. The MDA-MB-231 cell line is the most extensively studied breast cancer cell line, and evidence suggests that Nav1.5 and α-subunits are involved in the modulation of migration, invasion, proliferation, and chemosensitivity [32–41, 48–61]. Possible mechanisms of action include alteration in sodium currents, matrix metalloproteinase type 9 activity, Ki67 activity, and repressor element silencing transcription factor and histone deacetylases. Note that the Nav1.5 discovered in breast cancer is the neonatal form. The expression of neonatal Nav1.5 protein in breast cancer has been reported in relation to ERα status [42]. Neonatal Nav1.5 protein was not detected in the human brain, skeletal muscle, cardiac muscle, colon, small intestine, stomach, prostate, or bladder tissues [42]. The reason for the low-level neonatal Nav1.5 immunoreactivity observed in normal breast tissues remains unclear. Notably, neonatal Nav1.5 protein was significantly elevated in breast cancer compared to normal breast tissue, indicated by a two-fold increase in staining intensity and a 20-fold increase in the area of stained ductal structures [42]. These findings significantly build on cell- [32] and tissue-based studies [43]. The difference in the Nav1.5 expression pattern indicates that distinct cancer cells or distinct cell types activate various signaling pathways during growth, leading to the expression of different Nav channels on the cell membrane. Moreover, the pattern of neonatal Nav1.5 immunoreactivity in the plasma membrane became asymmetrical reflected by the increase in the apical/basal ratio value in the breast cancer. This is a noteworthy observation since cancer cells generally lose their polarity during metastasis [62]. Consequently, VGSC may exhibit unique behavior compared to other proteins in the plasma membrane, potentially due to its crucial role in the inherently directional metastatic process.

In the case of colon cancer, HCT116, HT29, SW480, and SW620 cell lines have been used, and they all express the Nav1.5 subtype [45–49]. It has been found that this subtype mediates cancer invasion by increasing sodium currents. Additionally, in SW480 and DLD1 cell lines, it has been suggested that Nav1.5 mediates proliferation, migration, and invasion, and increases chemosensitivity through the cell cycle, epithelial-mesenchymal transition, and Ras signaling pathways [45–49]. For ovarian cancer, the α-subunits may mediate proliferation and enhance chemosensitivity [50–52]. For endometrial cancer, primary cancer cells have been studied, and it has been found that Nav1.7 could mediate invasion and reduce apoptosis [53]. In prostate cancer cells PC3 α-subunits may mediate proliferation, migration, and invasion through the cell cycle and glucose uptake [54–60, 63]. Additionally, for gastric cancer, it has been found that Nav1.7 in BGC-823 and MKN-28 cell lines could mediate proliferation and invasion by regulating extracellular and intracellular pH via increased Na + /H + exchanger-1 [64]. Research into cervical cancer has suggested that Nav1.5, Nav1.6, Nav1.7, and β1-4 aid in the proliferation, migration, and invasion of SiHa, CaSki, and HeLa cell lines [51, 65–67]. Lung cancer studies have determined that Nav1.6 and Nav1.7 mediate invasion, and β1 and β3 are expressed in cancer cells using H23, H460, and Calu-1 cell lines [68–70]. NCI-H146 cell line has been used to investigate the effects of α-subunits on cancer cells. Interestingly, Nav1.7 has been found in thyroid cancer cell lines MZ-CRC-1 and TT, which mediates migration by its sodium currents [71, 72].

## In vivo evidence of VGSCs in cancers

Preclinical in vivo studies bearing on VGSCs in cancers collected from the published papers were summarized in Table 2. A number of different animal models have been used to study VGSCs in cancers, including mice and rat models. Both allograft and xenograft models have been applied, with most interference achieved either by VGSC knockdown in the cells used for modeling or by using VGSC inhibitors or activators [34, 46, 48, 51, 54, 60, 61, 64, 75–80]. Notably, a study using Pulsed Magnetic Field Stimulation to interfere with VGSC currents achieved quite promising results [81]. Similar to the in vitro studies, the most commonly studied cancer type is breast cancer [34, 48, 60, 61, 76, 77, 81], followed by prostate cancer [54, 78–80]. Most studies reported the expression of Nav1.5 and Nav1.7 VGSC subtypes in cancers, while the β-subunits were only reported in two studies, for β1 [34] and β3 [75] respectively. Most of the animal model studies implicated that the VGSCs mediated tumor growth with some suggesting also metastasis.

**Table 2:** Preclinical in vivo evidence of the impact of VGSCs on cancers

Cancer type	Model	Intervention	VGSC subtype	Effects of VGSCs	Reference
Breast	4T1-BALB/c mice c(allografts)	Intravenous Anti- Neonatal Nav1.5 Antibodies(inhibitor)	Nav1.5	Mediates metastasis	[48]
Breast	4T1-BALB/c mice (allografts)	Pulsed Magnetic Field Stimulation	α-subunits	Mediates tumor growth	[81]
Breast	MDA-MB-231-J/Nu mice (xenografts)	Pulsed Magnetic Field Stimulation	α-subunits	Mediates tumor growth	[81]
Breast	MDA-MB-231- Rag2/Il2rg Double Knockout mice(xenografts)	Intraperitoneal injection of phenytoin(inhibitor)	Nav1.5	Mediates tumor growth and metastasis	[60]
Breast	MDA-MB-231- Rag2/Il2rg Double Knockout mice(xenografts)	Nav1.5 knockdown in cells	Nav1.5	Mediates tumor growth and metastasis	[61]
Breast	MDA-MB-231- Rag2/Il2rg Double Knockout mice(xenografts)	Deletion of β1 in cells	β1	Mediates tumor growth, metastasis, and angiogenesis	[34]
Breast	MDA-MB-231-Luc- NMRI Nude Mice(xenografts)	Tail vein injection of Ranolazine(inhibitor)	Nav1.5	Mediates metastasis	[76]
Breast	DMBA-induced rat	Intraperitoneal injection of RS100642(inhibitor)	α-subunits	Mediates oxidative stress, affect survival	[77]
Colon	SW480-BALB/c mice(xenografts)	veratridine (activator) and tetrodotoxin (inhibitor)	Nav1.5	Mediates tumor growth	[46]
Prostate	Mat-LyLu-Copenhagen rat (allografts)	gavage ranolazine (inhibitor)	Nav1.7	Mediates metastasis	[78]
Prostate	Mat-LyLu-Copenhagen rat(allografts)	Subcutaneous injection of tetrodotoxin (inhibitor)	α-subunits	Mediates metastasis	[79]
Prostate	PC3-BALB/c mice(xenografts)	Subcutaneous injection of novel synthetic sodium channel blockers	α-subunits	Mediates tumor growth	[54]
Prostate	PC3-BALB/c mice(xenografts)	Intraperitoneal injection of racemic (inhibitor)	Nav1.7	Mediates tumor growth	[80]
Gastric	BGC-823- athymic mice(xenografts)	Nav1.7 knockdown in cells	Nav1.7	Mediates tumor growth	[64]
Ovarian	Caov-3-BALB/c mice(xenografts)	Intratumoral injection of a Nav1.5 antibody (inhibitor)or lidocaine (inhibitor)	Nav1.5	Mediates tumor growth	[51]
Liver	HepG2- male nude mice(xenografts)	β3 Nav1.7 knockdown in cells	β3	Mediates tumor growth	[75]

In breast cancer models such as 4T1-BALB/c mice and MDA-MB-231-J/Nu mice, interventions like intravenous administration of Anti-Neonatal Nav1.5 Antibodies [48] and Pulsed Magnetic Field Stimulation [81], directed respectively at Nav1.5 or pan-α-subunits, have emerged as strategies. These interventions have consistently demonstrated effects on tumor growth and metastasis, underscoring the significance of VGSCs in breast cancer progression [34, 60, 61, 76, 77]. Similarly, in prostate cancer models such as Mat-LyLu-Copenhagen rats and PC3-BALB/c mice, interventions including gavage ranolazine and subcutaneous injection of tetrodotoxin have shown promise in altering cancer progression by targeting Nav1.7 or pan-α-subunits, respectively [54, 78–80]. Furthermore, investigations into colon, gastric, ovarian, and liver cancer have utilized various interventions, from intratumoral injections of Nav1.5 antibodies to knockdown of β3 Nav1.7 in cells, reflecting the diverse approaches employed to modulate VGSCs across different cancer types [46, 51, 64, 75]. Overall, these experiments highlighted crucial insights into the therapeutic potential of targeting VGSCs in cancer treatment, underscoring their role in driving cancer metastasis and growth across a spectrum of malignancies.

## Clinical in vivo evidence of VGSCs in cancers

Clinical in vivo studies of VGSCs in cancers collected from the published papers are summarized in Table 3. In the clinical studies, colon cancer was the most prevalent [45, 46, 82–87], followed by breast cancer [32, 34, 42, 61, 82, 83] and prostate cancer [56, 60, 82, 83, 88]. Most studies focused on the α-subunits [32, 42, 45, 46, 52, 53, 56, 61, 64, 66, 73, 82–88], with only two reported β subunits [34] [60], where Nav1.5 was the most reported subtype [32, 42, 45, 52, 61, 73, 84, 85, 88]. For studies that determined the expression of VGSC in cancer tissue and compared it with normal tissue, almost all of them suggested overexpression of the α-subunits in cancers [32, 34, 42, 45, 52, 53, 56, 61, 64, 66, 73, 84–86, 88]. Eight studies suggested that VGSC was a risk factor for cancer [45, 53, 56, 64, 82, 83, 86], with four suggesting it was not associated with survival [34, 60, 61, 66]. Three of the studies involved VGSC inhibitors [82, 83, 87]. Despite the non-specificity, these studies interpreted the effects of VGSC inhibitors on cancer by proposing the role of VGSCs in cancers. These studies mostly did not specify the exact effect of VGSCs. The other studies suggested that VGSCs mediate metastasis or chemosensitivity.

**Table 3:** Clinical in vivo evidence of VGSCs in cancers

Cancer type	Sample size	VGSC subtype	Expression in cancer	Prognosis	Effects of VGSCs	Reference
Breast	59 528	α-subunits	/	Risk	VGSC drugs associated with survival	[82, 83]
Breast	496	Nav1.5	Overexpressed	/	Associates with Estrogen receptor-β expression	[42]
Breast	36	Nav1.5	Overexpressed	Not associated	/	[61]
Breast	40	β1	Overexpressed	Not associated	/	[34]
Breast	20	Nav1.5	Overexpressed	/	Mediates lymph node metastasis	[32]
prostate	50 601	α-subunits	/	Risk	VGSC drugs associated with survival	[82, 83]
prostate	160	Nav1.8	Overexpressed	Risk	/	[56]
prostate	15	β1-4	Not significant	Not associated	/	[60]
prostate	20	Nav1.2, Nav1.3, Nav1.5 and Nav1.6	Overexpressed	/	/	[88]
Gastric	18	Nav1.7	Overexpressed	Risk	/	[64]
Gastric	487	α-subunits	/	/	VGSC drugs associated with cancer	[87]
Colon (bowel)	22 867	α-subunits	/	Risk	VGSC drugs associated with survival	[82, 83]
Colon	136	Nav1.5	Overexpressed	Risk	/	[45]
Colon	182	Nav1.5	Overexpressed	Risk	/	[84]
Colon	497	Nav1.5	/	/	Enhances chemosensitivity	[46]
Colon	97	Nav1.1	Overexpressed	/	/	[85]
Colon	97	Nav1.6	Overexpressed	/	Mediates lymph node metastasis	[85]
Colon	269	Nav1.5	Overexpressed	Risk	Mediates lymph node metastasis, associates with Estrogen receptor-β expression	[86]
Colon	647	α-subunits	/	/	VGSC drugs associated with cancer	[87]
Lung	408	α-subunits	/	/	VGSC drugs associated with cancer	[87]
Haematological	299	α-subunits	/	/	VGSC drugs associated with cancer	[87]
Endometrial	80	Nav1.7	Overexpressed	Risk	Mediates metastasis	[53]
Cervical	57	Nav1.6	Overexpressed	Not associated	Mediates metastasis	[66]
Ovarian	53	Nav1.5	Overexpressed	/	/	[52]
Oral	8	Nav1.5	Overexpressed	/	/	[73]

Specifically, in breast, prostate, and colon cancer, VGSC-specific drugs have been associated with improved survival [45, 53, 56, 64, 82, 83, 86], suggesting that VGSCs may be involved in these cancers. In endometrial, cervical, and ovarian cancers, VGSCs have been found to increase metastasis [32, 53, 66, 85]. In addition, in breast and colon cancer, Nav1.5 has been found to be overexpressed [32, 42, 45, 61, 84], and this has been associated with an increased risk for cancer patients in colon cancer [45, 84, 86]. Lastly, in breast cancer, Nav1.5 has been found to mediate lymph node metastasis [32] and to associate with estrogen receptor-β expression [42].

## Insight from data reviewing

The data review included 9 α-subunit and 4 β-subunit of VGSC as shown in Fig. 1D and 33 types of cancers in The Cancer Genome Atlas (TCGA) as shown in S**-**Table 1 where the abbreviations were also listed. Data includes TCGA, Genotype-Tissue Expression (GTEx), and the Human Protein Atlas (HPA). In the transcriptomic analysis, a limitation is that several studies have reported the presence of the neonatal splice variant of Nav1.5 in cancer [32, 89, 90]. There is a difference of six amino acids between the neonatal Nav1.5 and normal Nav1.5, and the two splice variants have been shown to be pharmacologically distinct [89]. However, given that both variants are over 2000 amino acids long, using the adult Nav1.5 as a reference for sequencing and quantifying gene expression levels in this study might not result in any major differences.

### Single nucleotide variant (SNV) profile of VGSC in TCGA

**Fig. 2:**
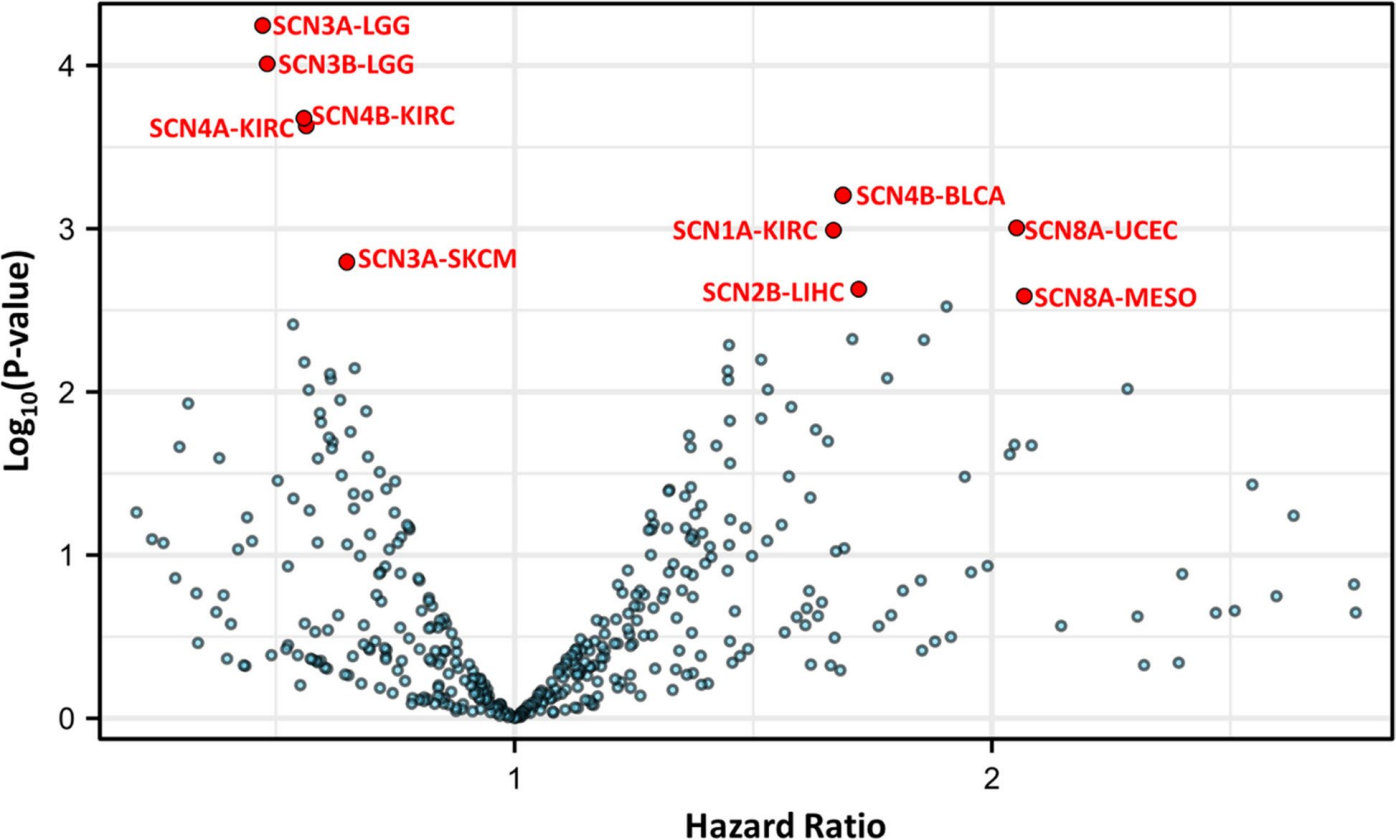
Survival association of VGSCs in cancers. The survival of patients of high and low expression (separated by median) in the cancer was compared. The volcano plot displays the hazard ratio of the VGSC gene-cancer type pair. Detail results were provided in S-Fig. 9 left panels

Based on analysis of TCGA data, the top five cancer types with mutations in VGSC genes (average rank in each cancer type) were skin cutaneous melanoma (SKCM), uterine corpus endometrial carcinoma (UCEC), colon adenocarcinoma (COAD), lung squamous cell carcinoma (LUSC), and stomach adenocarcinoma (STAD). The top 5 mutated VGSC genes in cancers are SCN1A, SCN9A, SCN11A, SCN2A, and SCN3A (S**-**Fig. 2A). The overall gene alteration frequency of both SCN1A and SCN10A is 22%, while that of SCN11A, SCN2A, and SCN5A is 20%. The most frequent variant classification is a missense mutation followed by a nonsense mutation and frameshift deletion. The most frequent variant type is a single nucleotide polymorphism (SNP). The commonest SNP class is C > T followed by C > A and T > C (S**-**Fig. 2B). However, all of the mutations occurred at a lower than 25% mutation frequency (S-Fig. 2A). In addition, survival analysis revealed that only a few VGSC mutations had a significant effect on overall survival, such as SCN3B in breast cancer (BRCA); however, this result should be interpreted with caution due to the low case number of the BRCA cohort, with only three cases of SCN3B mutation in BRCA (S**-** Fig. 2C).

VGSC α-subunits are indeed lengthy proteins with over 2,000 amino acid residues, which means they encompass a substantial number of structural elements where random mutations can occur. As a result, generally, they are more susceptible to a higher overall mutation rate compared to shorter proteins. The TCGA data suggested that the overall mutation rate of VGSC in cancers was relatively low. Uterine corpus endometrial carcinoma (UCEC) and skin cutaneous melanoma (SKCM) are the two cancer types with a few gene alteration frequencies greater than 20%. Despite this, the data from TCGA does not provide evidence to support the critical role of VGSC mutation in cancers. To date, no studies have reported a significant association between VGSC mutation and cancer, which is in line with the TCGA mining results.

### Expression profiles of VGSC in TCGA

This study compared the expression of VGSC in cancer and normal tissues across 33 cancer types from TCGA data. In the analysis of cancer-noncancer differences, two approaches were employed: (1) an unpaired t-test was used to compare cancer samples from TCGA with normal samples from both TCGA and the GTEx databases (S-Fig. 3); (2) a paired t-test was used to compare TCGA-paired cancer-normal samples from the same patient (S-Fig. 4). MESO and UVM were excluded from the first approach because there are no corresponding normal tissues for comparison. Thus, only the expression of cancer was displayed for MESO and UVM. The second approach excluded many cancer types due to the lack of paired normal samples. Additionally, a few cancer types had extremely low case numbers, such as SARC, SKCM, and THYM, and were only included for reference. The results of this analysis provide a systematic profile and important insights into the differences between cancer and normal tissue at the molecular level.

The Human Protein Atlas (HPA) database provides protein-staining data for SCN2A, SCN3A, SCN9A, SCN11A, and SCN4B. The reliability of the staining results was found to be limited due to the non-optimized experimental conditions and the undesirable properties of the antibodies. However, these data have been included in the present study for reference purposes. Representative images were displayed in S-Fig. 5. It should be noted that only the relevant gene-cancer data reported from literature studies have been presented, which facilitated for comparison at the end of this review.

Distinct cancer cells activate various signaling pathways during growth, leading to the expression of different Nav channels on the cell membrane. Currently, the field is focused on demonstrating the impact of Nav channels on cancer, rather than investigating the regulation of Nav channel gene expression. Therefore, the pathways or mechanisms regulating Nav channel gene expression in cancer cells remain unclear. To understand the regulation of VGSC in cancers, this study conducted an in-depth analysis to provide a systematic profile of the three expression-related aspects: copy number, methylation, and microRNAs (miRNAs). Specifically, the copy number variant (CNV) profiles of VGSC in cancers were displayed, presenting the percentage of different types of CNV and data of both heterozygous CNV and homozygous CNV (S-Fig. 6). Furthermore, the correlation of CNV/expression and correlation of methylation/expression were evaluated to explore the potential impact of CNV and methylation on expression (S-Fig. 7). In addition, miRNAs play a significant role in the regulation of cancer cells, influencing various aspects of cancer progression such as proliferation, invasion, and metastasis, potentially through the mediation of Nav channel expression. Here, we constructed the regulatory relationships and presented the miRNA-gene expression correlations in a network plot, based on data collected from databases. (S-Fig. 8). These profiles help to understand how VGSC expression is regulated in cancers and may be of great utility for future studies.

### Clinical association of VGSC in TCGA

The clinical association of each of the VGSC genes across 33 cancer types in TCGA was systematically analyzed in terms of survival association, immune subtype association, and molecular subtype association. The survival analysis, compared patients with high (above median) and low (below median) expression of the gene in the respective cancer type and calculated hazard ratios. Investigations of the immune and molecular subtype associations compared the expression levels of VGSCs between different subtypes. Detailed analysis results are presented in S-Fig. 9.

## Evidence comparison for key questions on VGSC in cancers

Bioinformatic evidence derived from high-throughput data and evidence from published data both offers insights, though both sources have their limitations. Therefore, it is prudent to combine and compare both to obtain a comprehensive understanding of the topic. A previous study applied similar principles to investigate the role of TRPM7 in cancer [1].

### Expression of VGSCs in cancers

VGSCs have been observed in many cancer cell lines (Table 4 column 14), providing a foundation for in vitro studies to explore the role of VGSCs in cancers. Although cell line models are very helpful for studying VGSC function in cancers, results from cancer and normal cell lines may not be directly comparable with tumor cells from clinical patients. Much as VGSCs have not been reported in some cancer types, the available studies suggest a greater VGSCs expression in cancer compared to normal tissues (Table 4 column 4). However, the TCGA data suggested a VGSC underexpression in tumor tissues of many cancer types, including colon cancer, prostate cancer, and breast cancer (Table 4 columns 6 and 7). The HPA protein staining data is generally in line with the TCGA data (Table 4 column 8).

**Table 4:** Summary evidence for VGSCs in cancers

1	2	3	4	5	6	7	8	9	10	11	12	13	14	15	16	17
			Clinical								Preclinical in vivo		In vitro			
Nav subtype	Gene	Cancer type	Expression in Cancer tissue from the published papers	Clinical Prognosis from the l published papers	Expression in Cancer tissue (TCGA + GTEx)	Expression in Cancer tissue (TCGA paired)	Protein expression (HPA)	Clinical Prognosis from TCGA	Immune subtype difference (TCGA)	Molecular subtype difference (TCGA)	Affect tumor growth in animal	Affect metastasis in animal	Expressed in cancer cells	Affect cancer cell proliferation	Affect cancer cell migration	Affect cancer cell invasion
α-subunits	/	Breast	/	Risky (1)	/	/	/	/	/	/	2	0	3	0	3	2
		Ovarian	/	/	/	/	/	/	/	/	0	0	1	1	0	0
		Prostate	/	Risky (1)	/	/	/	/	/	/	1	0	3	1	3	3
		Lung	Expressed (1)	/	/	/	/	/	/	/	0	0	1	0	0	0
		Colon	Expressed (2)	/	/	/	/	/	/	/	0	0	0	0	0	0
		Haematological	Expressed (1)	/	/	/	/	/	/	/	0	0	0	0	0	0
Nav1.1	SCN1A	Prostate	/	/	NS	NS	/	NS	NS	NS	0	0	1	0	0	0
		Colon	Overexpressed (1)	/	Underexpressed	Underexpressed	/	NS	NS	NS	0	0	0	0	0	0
Nav1.2	SCN2A	Prostate	Overexpressed (1)	/	Underexpressed	NS	Not detected	NS	NS	Yes	0	0	1	0	0	0
		Cervical	/	/	NS	NS	Overexpressed	Risky	NS	/	0	0	1	0	0	0
Nav1.3	SCN3A	Prostate	Overexpressed (1)	/	Underexpressed	Underexpressed	Underexpressed	NS	Yes	NS	0	0	0	0	0	0
Nav1.4	SCN4A	Cervical	/	/	Underexpressed	NS	/	NS	NS	/	0	0	1	0	0	0
Nav1.5	SCN5A	Colon	Overexpressed (3)	Risky (3)	Underexpressed	Underexpressed	/	NS	Yes	Yes	1	0	5	1	2	5
		Breast	Overexpressed (4)	Risky (1) or not associated (1)	Underexpressed	Underexpressed	/	Protective	Yes	Yes	1	3	19	4	14	13
		Ovarian	Overexpressed (1)	/	NS	/	/	NS	Yes	Yes	1	0	3	2	3	2
		Prostate	Overexpressed (1)	/	Underexpressed	Underexpressed	/	NS	Yes	NS	0	0	1	0	0	0
		Cervical	/	/	Underexpressed	NS	/	NS	Yes	/	0	0	1	1	1	1
		Oral	Overexpressed (1)	/	/	/	/	/	/	/	0	0	2	2	2	2
Nav1.6	SCN8A	Breast	/	/	NS	NS	/	NS	NS	Yes	0	0	1	0	1	0
		Prostate	Overexpressed (1)	/	Underexpressed	NS	/	NS	NS	Yes	0	0	3	1	1	1
		Cervical	Overexpressed (1)	Not associated (1)	NS	NS	/	NS	NS	/	0	0	2	0	0	1
		Lung	/	/	Overexpressed	Overexpressed	/	NS	Yes	Yes	0	0	1	0	0	1
		Thyroid	/	/	Overexpressed	/	/	NS	NS	/	0	0	1	1	0	1
Nav1.7	SCN9A	Breast	/	/	Underexpressed	Underexpressed	NS	NS	Yes	Yes	0	0	2	1	2	1
		Endometrial	Overexpressed (1)	Risky (1)	/	/	NS	/	/	/	0	0	1	0	0	1
		Prostate	/	/	Underexpressed	Underexpressed	NS	NS	Yes	Yes	1	1	5	1	3	2
		Cervical	/	/	Underexpressed	NS	Overexpressed	NS	NS	/	0	0	1	0	0	0
		Lung	/	/	Overexpressed	NS	Overexpressed	NS	Yes	Yes	0	0	2	0	0	2
		Gastric	Overexpressed (1)	/	NS	NS	Underexpressed	NS	Yes	Yes	1	0	0	0	0	0
		Thyroid	Expressed (1)	/	Overexpressed	Underexpressed	NS	NS	NS	/					1	
Nav1.8	SCN10A	Prostate	Overexpressed (1)	Risky (1)	NS	NS	/	NS	/	/	0	0	0	0	0	0
Nav1.9	SCN11A	Prostate	/	/	Underexpressed	NS	NS	NS	NS	NS	0	0	1	0	0	0
β1	SCN1B	Breast	Overexpressed (1)	Not associated (1)	Underexpressed	Underexpressed	/	NS	Yes	Yes	0	0	1	0	1	0
		Prostate	Expressed (1)	Not associated (1)	Underexpressed	Overexpressed	/	NS	NS	Yes	0	0	1	0	0	0
		Cervical	/	/	Underexpressed	NS	/	NS	Yes	/	0	0	2	1	1	0
		Lung	/	/	Underexpressed	Underexpressed	/	NS	Yes	Yes	0	0	1	0	0	0
β2	SCN2B	Prostate	Expressed (1)	Not associated (1)	Underexpressed	Underexpressed	/	NS	Yes	NS	0	0	1	0	0	0
		Cervical	/	/	Underexpressed	NS	/	NS	NS	/	0	0	1	0	1	0
β3	SCN3B	Prostate	Expressed (1)	Not associated (1)	Underexpressed	Underexpressed	/	NS	Yes	Yes	0	0	1	0	0	0
		Cervical	/	/	Underexpressed	NS	/	NS	NS	/	0	0	1	0	1	0
		Lung	/	/	Underexpressed	Underexpressed	/	NS	Yes	NS	0	0	1	0	0	0
		Liver	/	/	Overexpressed	NS	/	NS	NS	NS	1	0	1	1	0	0
β4	SCN4B	Prostate	Expressed (1)	Not associated (1)	Underexpressed	Underexpressed	Underexpressed	NS	Yes	Yes	0	0	1	0	0	0
		Cervical	/	/	Underexpressed	NS	Underexpressed	NS	Yes	/	0	0	2	0	1	1

The number or number in “()” represents the number of pieces of evidence. “/”represents no data available. NS represents not significant

Such inconsistencies might result from biases in pre-hypothesis studies from the experiments or inherent biases in the TCGA bulk sequencing data. A major issue with much of the TCGA data is that it relies on bulk RNAseq, while the tumor microenvironment is highly heterogeneous. For example, VGSCs may only be expressed in a small subpopulation of cells within the tumor microenvironment, resulting in 'low' overall expression in the bulk sample. In certain cancer types, a notable limitation arises due to the potential incongruence between the origins of normal tissue and the tumor tissue. For example, in the context of glioma, the comparison between cancer and normal tissues in this study might be inaccurately portrayed. The normal bulk sample utilized encompasses brain tissue more broadly, rather than specifically targeting glial cells where glioma is derived. It is essential to emphasize that the majority of cells in brain tissue are neurons, and these neurons may express high levels of VGSCs. This expression pattern within neurons could potentially introduce a confounding factor, influencing the accuracy and interpretation of the analysis.

Another issue with TCGA data is that it only provides an indication of transcript levels, missing any post-transcriptional or post-translational regulation of these ion channels. Therefore, the channels may be present and functional even if bulk transcript levels are relatively low. Emerging technologies, such as single-cell RNAseq and spatial transcriptomics, will hopefully help resolve these contradictions in the future. Nevertheless, the aberrant expression levels of VGSCs in some cancer types in the RNA sequencing data, whether overexpressed or underexpressed, support their potentially different roles in cancer and normal tissues, and they could still be potential clinical diagnostic biomarkers with RNA sequencing.

### Survival associations of VGSCs in cancers

Expression and survival are two of the most fundamental pieces of information provided by TCGA. Despite a few studies that have reported the risky prognostic effects of VGSCs in cancer(Table 4 column 5), the TCGA data suggests that the most commonly studied VGSC genes do not associate with patient survival (Table 4 column 9). The inconsistency between the published papers and TCGA reflects the potential bias in the VGSC-cancer studies. Notably, the SCN5A gene, the most studied VGSC in breast cancer, was reported as a risk factor in one study and not associated with altered survival in another study. However, the TCGA data suggests that SCN5A is associated with improved survival in TCGA breast cancer. As shown in the Kaplan–Meier Plots, TCGA data suggests that SCN5A is slightly associated with better overall survival, disease-specific survival, and progress-free interval. The GEO data (accessed from the Kaplan–Meier Plotter) validated that SCN5A is associated with better overall survival and progress-free interval [91]. The GEO data also suggested that better post-progression survival is also associated with high SCN5A [91]. However, the higher SCN5A expression is associated with distant metastasis-free survival in breast cancer [91]. This clinical data indicates that SCN5A might contribute to breast cancer metastasis, and this conclusion aligns with many in vitro studies.

### Functional impact of VGSCs on cancer cells

Preclinical studies in vitro and in vivo have provided functional evidence for the involvement of VGSCs in cancer cell proliferation, migration, and invasion (Table 4 columns 15, 16, and 17). The most studied VGSC, Nav1.5, has been studied in the context of breast and colon cancers. More than 12 papers with in vitro studies on Nav1.5 in breast cancers suggested the channel could facilitate cell migration and invasion, with four studies additionally reporting increased cancer cell proliferation. The role of Nav1.5 in breast cancer cell migration and invasion in vitro has been observed to correlate with the results of a clinical distant metastasis-free survival analysis in breast cancer from GEO data (accessed from the Kaplan–Meier Plotter) (S-Fig. 10). In colon cancer, Nav1.5 was also shown to enhance the invasion of cancer cells. Although most in vitro studies suggested that the VGSC α-subunit functions in cell migration, preclinical in vivo studies have suggested that the α-subunit of VGSC can facilitate tumor growth, with a few also suggesting it could facilitate metastasis. Fewer studies have been conducted on VGSC β-subunits; these have indicated that the β1 subunit could affect cervical cancer cell proliferation and that the β4 subunit could affect invasion. Additionally, the β3 subunits were reported to potentially affect cancer cell apoptosis in liver cancer. However, despite preclinical evidence suggesting a promising role of VGSCs in cancer, caution should be exercised in interpreting the implications of these data, because, as mentioned, large-scale datasets TCGA indicate that VGSC expression is generally lower in cancer than in normal tissues and that the expression of VGSCs generally does not associate with patient survival. Given the bias in RNA sequencing data, it remains unclear if these functional impacts of VGSCs on cancer cells could make a clinically meaningful difference in cancer patients. Nevertheless, One consistent result between experimental evidence and clinical evidence is that VGSCs can impact cancer migration.

## Possible mechanisms underlying the impact of VGSCs on cancer

### Persistent sodium current

Although membrane potentials in cancer cells can be dynamic and oscillating [92], the resting membrane potential of cancer cells has been reported to range from -5 to -52 mV, and that of highly proliferating non-cancer cells falls within the range of -5 to -25 mV [15]. By contrast, in non-cancer cells, the resting membrane potential is typically between -95 and -40 mV [15]. In the presence of Nav channels, this might result in a larger persistent sodium current, leading to downstream sodium gradient cascades and activating various signaling pathways and sodium-associated transmembrane mechanisms, such as Na + /H + exchangers [45] and sodium/calcium exchanger [93].

An exciting progression in the field is the identification of ranolazine, a specific blocker for sodium channel persistent current in cancer cells [94], as a clinically viable anti-metastatic drug that improves cancer survival [76, 78, 95]. Interestingly, in contrast to ranolazine, sodium channel blockers such as lidocaine, that inhibit peak current do not enhance cancer survival [82, 83]. These studies, highlighting the distinct roles between sodium peak current and sodium persistent current in cancer [82] should be given more prominence.

### Sodium and hydrogen

The sodium–hydrogen antiporter 1 (sodium–proton exchanger proteins, NHE1) is co-expressed with VGSCs and can increase intracellular alkalization and extracellular acidity. The acidic microenvironment of cancer cells promotes the degradation of the extracellular matrix by cysteine cathepsins [96], thereby facilitating cancer migration and invasion [38, 97, 98]. An allosteric interaction between Nav1.5 and NHE1 has been suggested to explain a Nav1.5-dependent increase in H^+^ extrusion by NHE1 [39]. For the cardiac subtype VGSC, Nav1.5, another additional possibility is that Na^+^ influx through Nav1.5, rather than the Nav1.5 protein itself, increases H^+^ extrusion through NHE1 and other pH regulators, thereby resulting in extracellular acidification [39]. The extracellular acidification facilitate the invasion of cells [45]. Moreover, low pH can positively regulate Nav1.5 function in cardiomyocytes by increasing the persistent Nav current carried by Nav1.5 [99, 100], which might also occur in cancer cells.

### Sodium and calcium

Although the whole-cell Ca^2+^ release-activated Ca^2+^ current was reported to be independent of extracellular and cytosolic Na^+^ [101], sodium/calcium exchanger, a unique calcium transport system that typically exports calcium ions out of the cell in exchange for sodium ions [102], might play a role in tumors [103, 104]. The sodium/calcium exchanger is another energetically unfavorable physiological processes that known to be driven by sodium gradients [93]. The sodium/calcium exchanger facilitates the movement of sodium down its concentration gradient and calcium in the opposite direction. Typically, sodium, which is at a higher concentration in the extracellular matrix (ECM), is transported into the cell, while calcium is moved out. However, sodium/calcium exchanger can also operate in reverse mode, bringing calcium into the cell. Calcium ions are crucial for numerous physiological processes, including vesicle transport and exocytosis [105], signal transduction as secondary messengers [105], muscle contraction [106], and as cofactors in various biological reactions. A rapid influx of sodium ions through voltage-gated sodium channels (VGSC) might affect sodium/calcium exchanger activity, subsequently altering calcium handling for processes such as vesicle exocytosis or signal transduction in invadopodia. Data also suggested that sodium ion influx can activate intracellular calcium signaling pathway [107]. This increases the uptake of calcium ions by mitochondria and further leads to their release of calcium ions into the cytosol [108]. Higher calcium concentrations in the cytosol promote the formation of invadopodia, facilitating cell movement [109, 110]. This hypothesis is largely based on the observation of VGSCs in macrophage and microglial podosomes [111] but might contribute to cancer cell migration.

### β subunits

As immunoglobulin (Ig) family cell-adhesion molecules, VGSC β subunits are proposed to regulate cell adhesion, but some studies report that β subunit subtypes regulate cancer migration and invasion in a range of different ways. In breast cancer cells, β1 expression was negatively associated with cancer metastasis [112], while in prostate cancer, overexpression of β2 was associated with an increase in cancer migration and invasion [113]. The expression of β4 was reported to be downregulated in breast cancer cells compared to that in non-cancer epithelial cells. Reduced β4 expression was reported to promote migration and invasion while overexpressed β4 did the opposite [114]. β3 expression was absent in two breast cancer cell lines [112], but took place in other cancers such as prostate cancer [60] and liver cancer [115]. A recent study revealed that β3 can bind to tumor suppressor p53 and facilitate the degradation of p53 protein in liver cancer [115]. Although some effects of the β subunit on cancers have been reported, the underlying mechanisms remain largely unknown.

### Growth factor

VGSCs have been suggested to be involved in growth factor regulation in cancers. Epidermal growth factor (EGF) was reported to promote the migration and invasion of prostate and non-small cell lung cancer cells by increasing Nav1.7 expression [59, 68, 116]. The regulatory role of nerve growth factor (NGF) in prostate cancer was also found to be associated with the up-regulation of Nav1.7 [117, 118]. Furthermore, some growth factors that are critical in cancers have been found to interact with VGSCs in non-cancer cells. For example, vascular endothelial growth factor (VEGF), a key regulator for cancer angiogenesis [119], has been found to increase VGSC expression in the DRG neurons [120]. However, another critical regulator in cancer, transforming growth factor-beta 1 (TGF-β1) has a paradoxical role in cancers [121] and was upregulated when Nav1.5 was inhibited in cardiac myocytes and fibroblasts [122]. These inferred that the VGSCs might not necessarily provide growth signals directly through growth factors, but are involved in more complex regulatory mechanisms.

### Hormones

A number of studies have also shown that VGSCs are closely associated with the secretion of hormones, that are critical for some cancer types such as breast cancer and prostate cancer. In cardiomyocytes, insulin response elements in the SCN5A promoter region can affect the expression of Nav1.5 [123]. In adrenal chromaffin cells and breast cancer cells, insulin was also reported to regulate VGSC expression [124, 125]. Interestingly, the expression of functional VGSCs was found to be potentially associated with the expression of estrogen receptors (ERs) in breast cancer cells [32] and the expression of androgen receptors (ARs) in prostate cancer cells [126, 127].

## VGSC-targeting drugs for cancers

In previous studies concerning VGSCs in the context of cancer, several VGSC-targeting drugs have been employed for research in this field. Table 5 provides a summary of VGSC-targeting drugs used in cancer studies as reported in the published papers. Not surprisingly, Tetrodotoxin, the most classic VGSC blocker, is used in many studies. Local anesthetics, which primarily target and inhibit VGSCs, have also been widely utilized in VGSC-cancer studies [49–51, 128]. Additionally, antibodies [48, 51], toxins [32, 37, 41, 45, 48, 50, 52, 53, 56, 57, 59, 67–70, 74, 76, 78, 79, 94, 127, 129, 130], chemical small molecules [45], and natural products [51, 67, 131] have been applied in VGSC-cancer studies. This summary of drug effects and doses provides a reference for future studies targeting VGSC in cancer and serves as a guide for locating relevant studies.

**Table 5:** VGSC-targeting drugs for cancers

Drug	Cancer type	Cell lines	VGSC subtype	Effects on VGSCs	Effects on cancer cells	Dose	Reference
Tetrodotoxin	Colon, breast, lung, Prostate, Ovarian, lung, oral	HCT116, HT29, SW480 and SW620; MDA-MB-231, MCF-7, and 4T1	Nav1.5	Decreases sodium currents	Reduce migration and invasion	10–30 µM	[32, 37, 41, 45, 48, 50, 52, 53, 57, 59, 67–70, 74, 79]
Phenytoin	Breast	MDA-MB-231 and MCF-7	Nav1.5 and Nav1.7	Decreases sodium currents	Reduce proliferation, migration, and invasion	50 µM	[33, 59, 60]
3D-QSAR model-design Small-molecule Nav1.5 inhibitors	Colon	HCT116, HT29, SW480 and SW620	Nav1.5	Decreases sodium currents	Reduce invasion	5–30 µM	[45]
Polyclonal and monoclonal antibodies to Nav1.5	Breast	MDA-MB-231 and 4T1	Nav1.5	/	Reduce invasion	10–60 µg/ml	[48]
α-Hydroxy-α-phenylamides	Prostate	PC3	Nav1.7	Decreases sodium currents	reduce the size of tumors	10 mg/kg	[80]
Local anaesthetics	Ovarian	KURAMOCHI, OVCAR3, OVCAR5, JHOS4, and OVSAHO	α-subunits	Decreases sodium currents	Reduce proliferation	1–10 mM	[50]
Lidocaine	Ovarian	Caov-3	Nav1.5	Decreases sodium currents	Reduce proliferation	1 mM	[51]
Ropivacaine	Colon	SW620	Nav1.5	Decreases sodium currents	Reduce invasion	3.8 µM	[49]
Eicosapentaenoic acid	Ovarian	TOV112D, A2780 and SKOV3	Nav1.5	Decreases sodium currents	Reduce proliferation and migration	200µM	[51]
Hydroxyamides drugs	Prostate	PC3	/	Decreases sodium currents	Reduce proliferation	50µM	[127]
Hydantoin drug	Prostate	PC3	/	Decreases sodium currents	Reduce proliferation	50µM	[127]
Imipridone TIC10	/	HEK293	Nav1.5	Decreases sodium currents	/	4 µM	[132]
Oleuropein	Prostate	MAT-LyLu	Nav1.7	Decreases expression	Reduce proliferation and migration	250 μg/mL	[133]
PF-05089771	Endometrial	Primary endometrial cancer cell	Nav1.7	Decreases sodium currents	Reduce invasion, enhances apoptosis	100µM	[53]
Propranolol	Breast	MDA-MB-231	Nav1.5	Decreases sodium currents	Reduce migration and invasion	25 μM	[94]
Eicosapentaenoic acid	Prostate	PC-3 and Mat-LyLu	Nav1.6 and Nav1.7	Decreases expression	Decreases proliferation, migration, and invasion	30 μM	[58]
AMTB	Breast	MDA-MB-231 and SK-BR-3	NaV1.5	Decreases sodium currents	Reduce proliferation and migration	100µM	[54]
Docosahexaenoic acid	cervical	Primary cancer cell	α-subunits	Decreases expression	Reduce migration	0.5 µM	[67]
RS100642	Breast	DMBA-induced rat tumor	α-subunits	Decreases sodium currents	Reduce oxidative Stress	0.25 mg/kg	[77]
FS50 (protein from the animal)	Breast	MDA-MB-231	Nav1.5	Decreases expression	Reduce migration	10 μM	[52]
S0154	Prostate	PC3, DU145, and LnCaP	α-subunits	Decreases sodium currents	Decreases proliferation, migration, and invasion	10 μM	[54]
S0161	Prostate	PC3, DU145, and LnCaP	α-subunits	Decreases sodium currents	Decreases proliferation, migration, and invasion	10 μM	[54]
Caffeic acid phenethyl ester	Breast	MDA-MB-231 and MDA-MB-468	α-subunits	Decreases sodium currents	Reduce migration	1 μM	[131]
Naringenin	Prostate	MAT-LyLu	Nav1.7	Decreases expression	Decreases migration and invasion	10 μM	[57]
Ranolazine	Breast, prostate, colon	MDA-MB-231, Mat-LyLu, SW620	Nav1.5 Nav1.7	Decreases sodium currents	Decreases migration and invasion	5 μM	[48, 76, 78, 94, 95]
Lambert-Eaton syndrome IgG	Lung	NCI-H146	α-subunits	Decreases sodium currents	/	/	[70]
E3Ab (antibody)	Cervical, breast, ovarian	SiHa, MDA-MB-231, and Caov-3	Nav1.5	Decreases sodium currents	Decreases proliferation, migration, and invasion	15 µg/mL	[51]
AaH-IV (toxin)	Prostate	DU145	Nav1.6	Increases sodium currents	Decreases proliferation	/	[129]
JZTX-I (toxin)	Prostate	Mat-LyLu	Nav1.7	Increases sodium currents	Enhances migration and invasion	5 μM	[130]
HNTX-III (toxin)	Prostate	Mat-LyLu	Nav1.7	Decreases sodium currents	Reduce migration and invasion	5 μM	[130]
SV188	Thyroid	MZ-CRC-1 and TT	Nav1.7	Decreases sodium currents	Reduce migration	3 μM	[72]
Trichostatin A	Breast	MDA-MB-231 and MCF-7	Nav1.5	Increases expression	Decreases proliferation, enhances migration, and invasion	1 µg/mL	[55]
JZTX-14 (toxin)	Breast	MDA-MB-231	Nav1.5	Decreases sodium currents	Decreases migration and invasion	6 μM	[56]

However, there is a limitation in the field due to the non-specific nature of many drugs targeting Nav channels. Although some of these drugs might preferentially inhibit certain subtypes of Nav channels (such as TTX – see Introduction), their application is generally not specific to a single subtype but has a broad effect on multiple Nav channel subtypes. This lack of specificity may be overlooked by many studies, as most do not suggest the role of a single Nav channel subtype in cancer, but rather focus on the general Na currents that all subtypes can mediate [32, 37, 41, 45, 48, 50, 52, 53, 57, 59, 67–70, 74, 79]. For example, sodium current-targeting nerve growth factor was identified for prostate cancer cell lines, without distinguishing among subtypes [117]. These pan-VGSC drugs could lead to non-specific effects on normal tissues, resulting in side effects that prevent these candidate drugs from progressing from in vitro studies to clinical application. Hence, it is essential to identify subtype-specific drugs to achieve cancer-specific treatment. An attempt to target neonatal Nav1.5 has generated a specific antibody against an epitope that is unique to neonatal Nav1.5, thus aiming to specifically target cancer [48]. Here, we urge the future development of more VGSC subtype-specific targeting strategies to achieve cancer-specific treatment.

## VGSC and cancer drug resistance

As discussed, one of the most plausible mechanisms through which VGSCs impact cancer is by potential regulation of cancer cell migration. As the β subunit is less studied, this discussion focuses on the α subunits. Many studies have suggested that VGSCs may modulate migration by influencing the epithelial-to-mesenchymal transition (EMT) phenotype [46, 49, 50]. EMT has been linked to therapy resistance in many cancer types, such as lung cancer [134, 135], pancreatic cancer [136], and breast cancer [137, 138]. Therefore, the inhibition of VGSCs, hindering EMT, could represent a pathway through which VGSCs are involved in drug resistance.

In a prior study, a hypothesis was proposed suggesting that intervening with VGSCs could potentially overcome drug resistance in cancer [139]. According to this hypothesis, the inhibition of VGSCs has the potential to impede both EMT and angiogenesis through interactions with intracellular calcium activity and endothelial cells, respectively. Combining the blockage of VGSCs with other anticancer therapies may prove effective in both adjuvant and palliative settings. The inhibition of VGSCs might slow down the colonization at secondary sites by hindering angiogenesis, thereby providing temporary relief from symptoms associated with the tumor burden in patients with metastatic disease [139].

The VGSC inhibitors with the potential to inhibit EMT, could be particularly efficacious in the adjuvant setting. Disseminated and circulating tumor cells that have undergone EMT tend to be less proliferative, rendering them less responsive to chemotherapy. Inhibiting EMT may disrupt dormancy and enhance the chemosensitivity of cells, as observed with valproic acid in glioblastoma [140]. Cells in the disseminated and circulating tumor state exhibit mesenchymal characteristics due to EMT. Following the transition to a mesenchymal phenotype, cellular dependence on EGFR signaling diminishes, activating alternative growth factor pathways [141]. The reduction in EGFR expression during mesenchymal transition may explain the limited efficacy of incorporating anti-EGFR agents like cetuximab into chemotherapy in the adjuvant setting [142]. In addition, the epidermal growth factor was reported to increase Nav1.7 expression [59, 68, 116], which might be potentially involved in this regulatory pathway of drug resistance.

To date, only a limited number of experimental studies have delved into the role of VGSC in cancer drug resistance. Among these investigations, a study centered on leukemia has uncovered a direct association between VGSC and drug resistance in this context [143]. Specifically, this study has linked the augmentation of the voltage-gated sodium current to multidrug resistance in leukemia cells. Employing a patch clamp technique, the study measured the voltage-gated sodium current in a drug-sensitive human leukemia cell line, K562, and its multidrug-resistant counterpart (resistant to anthracycline antibiotics and Vinca alkaloids). The results indicated that a significant proportion of the multidrug-resistant cells exhibited voltage-gated sodium current, contrasting with the predominant absence of such current in the parental drug-sensitive cells. Unfortunately, doubts arose when tetrodotoxin failed to restore sensitivity to doxorubicin and vincristine, challenging the established link between drug resistance and VGSC [143].

Another study in the context of ovarian cancer reported that a VGSC-targeting drug, lidocaine, hinders the metastatic capabilities of ovarian cancer by impeding Nav1.5-mediated EMT and the focal adhesion kinase/Paxillin signaling pathway [144]. Elevated focal adhesion kinase levels were observed in advanced-stage ovarian cancers and correlated with advanced drug resistance to platinum- and taxane-based chemotherapy in ovarian cancer patients [145, 146]. In this study, when ovarian cancer cells were treated with 10 μM cisplatin combined with 5 mM lidocaine, cell viability decreased by 40% compared to cells treated with cisplatin alone. The combination of lidocaine and cisplatin enhanced the deactivation of the focal adhesion kinase/Paxillin signaling pathway and the induction of apoptosis compared to the effects observed with cisplatin alone [144]. In vivo experiments corroborated these findings, showing that the combined administration of lidocaine and cisplatin significantly decreased ovarian cancer loading. This combination exhibited superior inhibitory effects on cancer malignancy compared to individual drug treatments [144]. Besides the focal adhesion kinase/Paxillin signaling pathway, this study also attributed the effect of VGSC on drug resistance to the induction of apoptosis by the VGSC [144]. This finding aligns with another investigation indicating that Nav1.5 augments 5-Fluorouracil-stimulated apoptosis in colorectal cancer cells [46]. The study showed that stage II/III colorectal cancer patients with upregulated SCN5A expression demonstrated enhanced survival after 5-Fluorouracil-based adjuvant chemotherapy. In vitro experiments further suggested that SCN5A knockdown increased the IC50 for 5-Fluorouracil by elevating 5-Fluorouracil-induced apoptosis [46].

## Future studies

The aim of this work was to identify potential new and unexplored scientific questions that could represent avenues for future research in this field. By doing so, we hope to expand our understanding of the subject and spur further exploration of its possibilities. To this end, we have identified gaps or areas that have yet to be explored based on the current published papers and our data reviewing. It is important to note that the majority of VGSC subtypes have never been studied in the context of cancer. This is due to the fact that some VGSCs are not expressed, or are expressed at extremely low levels, in some cancer types. Additionally, researchers often focus on one subtype of VGSCs and ignore the other subtypes in their studies. For example, many studies apply inhibitors that are not subtype-specific to VGSCs but attribute the effect to only one subtype.

A significant research area for future studies would be the investigation of the role of VGSCs in certain cancer types with potential clinical impact. Specifically, it is important to investigate VGSCs in cancer types where their gene expression is relatively high, in order to develop applicable biomarkers for clinical use. Furthermore, the expression or otherwise of the gene should have an impact on patient survival, as this would demonstrate that it is making a considerable difference in real patients. Additionally, it would be preferable (but not essential) to consider VGSCs that are aberrantly expressed in cancer tissue compared to normal tissue, as this could provide potential cancer-specific drug targets for treatment. To ensure reliability, such analysis should be conducted with a large sample size. To propose potentially significant research topics for future studies, the information on the top significant gene-cancer pairs from TCGA was displayed in Fig. 2 and S**-** Table 2. Among these gene-cancer pairs, SCN3A-LGG, SCN3B-LGG, SCN4A-KIRC, SCN1B-UVM, and SCN1B-PAAD have a relatively high gene expression level, and have a relatively large case number except for UVM (*n* = 79). No studies to date have investigated the potential connections between these gene-cancer pairs, therefore, SCN3A-LGG, SCN3B-LGG, SCN4A-KIRC, and SCN1B-PAAD would be potential clinically significant research topics for future study.

Another aspect of the potential study in this field in the future is to investigate the potential of VGSCs to be used for cancer therapies. Our bioinformatic analysis suggested a potential association between VGSC expression and the immune subtypes in certain cancer types (Table 4 column 10), hinting at the possibility of using VGSCs as a prediction biomarker or enhancer in cancer immunotherapy, such as Nav1.5 in colon, breast, and ovarian cancer and Nav1.7 in breast, prostate, lung, and gastric cancer. However, this analysis can provide some hints but not sufficient evidence to support the role of VGSCs in the cancer immune environment. In fact, a number of bioinformatic studies have used TCGA data for immune cell infiltration analysis and immune association studies of genes, but these bulk RNA sequencing analyses provide associations rather than causal effects of a gene on cancers [27–30, 147–152]. Emerging technologies, such as single-cell RNA sequencing and spatial transcriptomics, will hopefully offer more insights into the interactions between cancer cells and immune cells in the future, and reveal whether VGSCs play a role in this communication.

Additionally, there is already in vitro data available that demonstrates the direct inhibition of VGSC blockers on cancer cells, suggesting that the development of VGSC blockers as chemotherapy or chemotherapy enhancers is promising. Our analysis also suggested that VGSC levels are associated with the molecular subtype of some cancer types (Table 4 column 11), implying that VGSC-targeting cancer drugs could be tailored to specific molecular subtypes. As summarized in this study, many VGSC-targeting drugs are readily accessible for research in this area, promising major future advances in this field. However, given the VGSC subtype similarity, specifically targeting VGSC subtypes has proven to be extremely difficult. Many current studies treat pan-VGSC as a single entity, which can lead to non-specific effects on normal tissues. Therefore, developing subtype-specific therapies is essential, as demonstrated by pioneering work on neonatal Nav1.5 [89]. We urge further research in this area to achieve more precise and effective treatments.

Almost all studies in this field so far focus on the downstream effects of VGSCs, demonstrating how VGSCs impact downstream functions in cancer. However, there is a lack of investigation into the upstream regulation of VGSC expression. A recent study proposed a hypothesis suggesting the presence of a feedback loop in Nav1.5-mediated cellular invasion that regulates the expression of Nav1.5 in cancer cells [153]. We emphasize the need for further research to validate these mechanisms. Given the importance of VGSC expression in cancer, additional research is essential to understand and validate the upstream pathways regulating VGSC expression. This study provides new insights and indicates upstream regulation of VGSC expression using open databases in aspects of copy number, methylation, and miRNAs. Hopefully, these pieces of information can inspire interest and highlight potential research candidates for future studies. This could also potentially lead to the identification of drug targets that mediate VGSC expression rather than merely blocking the channels and uncover the intrinsic relationships between VGSCs and other correlated oncogenes.

### Supplementary Information

Supplementary Material 1.

## Data Availability

No datasets were generated or analysed during the current study.
