# Supplementary material for: Voltage-gated sodium channels in cancers
Source: Biomark Res. 2024 Jul 25;12:70. doi: 10.1186/s40364-024-00620-x (PMC11282680; doi:10.1186/s40364-024-00620-x)
Supplement: Supplementary file 1 — Supplementary Material 1. [file 40364_2024_620_MOESM1_ESM.docx]

**Supplementary materials**

**S-Table 1.** Abbreviations of cancer types in TCGA

| Abbreviations | Full names |
| --- | --- |
| ACC | Adrenocortical carcinoma |
| BLCA | Bladder Urothelial Carcinoma |
| BRCA | Breast invasive carcinoma |
| CESC | Cervical squamous cell carcinoma and endocervical adenocarcinoma |
| CHOL | Cholangio carcinoma |
| COAD | Colon adenocarcinoma |
| DLBC | Lymphoid Neoplasm Diffuse Large B-cell Lymphoma |
| ESCA | Esophageal carcinoma |
| GBM | Glioblastoma multiforme |
| HNSC | Head and Neck squamous cell carcinoma |
| KICH | Kidney Chromophobe |
| KIRC | Kidney renal clear cell carcinoma |
| KIRP | Kidney renal papillary cell carcinoma |
| LAML | Acute Myeloid Leukemia |
| LGG | Brain Lower Grade Glioma |
| LIHC | Liver hepatocellular carcinoma |
| LUAD | Lung adenocarcinoma |
| LUSC | Lung squamous cell carcinoma |
| MESO | Mesothelioma |
| OV | Ovarian serous cystadenocarcinoma |
| PAAD | Pancreatic adenocarcinoma |
| PCPG | Pheochromocytoma and Paraganglioma |
| PRAD | Prostate adenocarcinoma |
| READ | Rectum adenocarcinoma |
| SARC | Sarcoma |
| SKCM | Skin Cutaneous Melanoma |
| STAD | Stomach adenocarcinoma |
| TGCT | Testicular Germ Cell Tumors |
| THCA | Thyroid carcinoma |
| THYM | Thymoma |
| UCEC | Uterine Corpus Endometrial Carcinoma |
| UCS | Uterine Carcinosarcoma |
| UVM | Uveal Melanoma |

**S-Table 2.** The top 20 most significantly survival-associated VGSC genes-cancer pair in TCGA.

| Gene-cancer | Average expression Log_2_(TPM+1) | Fold change (compared to normal) | Hazard ratio (HR) | HR(p-value) | N | Studied previously? |
| --- | --- | --- | --- | --- | --- | --- |
| SCN3A-LGG | 3.77 | 1.66 | 0.47 | 5.60E-05 | 523 | No |
| SCN3B-LGG | 3.95 | 1.05 | 0.48 | 9.79E-05 | 523 | No |
| SCN4B-KIRC | 0.29 | 1.85 | 0.56 | 0.000209503 | 531 | No |
| SCN4A-KIRC | 1.12 | 2.09 | 0.56 | 0.000221016 | 531 | No |
| SCN4B-UVM | 0.04 | / | 7.21 | 0.000351531 | 79 | No |
| SCN1B-UVM | 3.00 | / | 6.71 | 0.000608547 | 79 | No |
| SCN4B-BLCA | 0.06 | 0.40 | 1.69 | 0.000637795 | 407 | No |
| SCN8A-UCEC | 0.30 | 0.63 | 2.05 | 0.001008804 | 181 | No |
| SCN1A-KIRC | 0.29 | 2.19 | 1.67 | 0.001050019 | 531 | No |
| SCN1A-ACC | 0.08 | 0.79 | 3.90 | 0.001442089 | 77 | No |
| SCN3A-SKCM | 0.13 | 0.49 | 0.65 | 0.001614292 | 469 | No |
| SCN2B-LIHC | 0.02 | 1.07 | 1.72 | 0.002297472 | 371 | No |
| SCN8A-MESO | 0.96 | / | 2.07 | 0.002656488 | 88 | No |
| SCN2A-UCEC | 0.23 | 0.38 | 1.91 | 0.002999269 | 181 | No |
| SCN1B-PAAD | 3.26 | 1.21 | 0.54 | 0.003865157 | 179 | Yes |
| SCN9A-GBM | 0.55 | 0.79 | 1.71 | 0.004753415 | 166 | No |
| SCN4A-UCEC | 0.13 | 0.58 | 1.86 | 0.004804497 | 181 | No |
| SCN4B-OV | 0.07 | 1.38 | 1.45 | 0.005168884 | 427 | No |
| SCN3B-BLCA | 0.16 | 0.25 | 1.52 | 0.006349437 | 407 | No |
| SCN11A-PAAD | 0.10 | 1.28 | 0.56 | 0.006586887 | 179 | Yes |

**
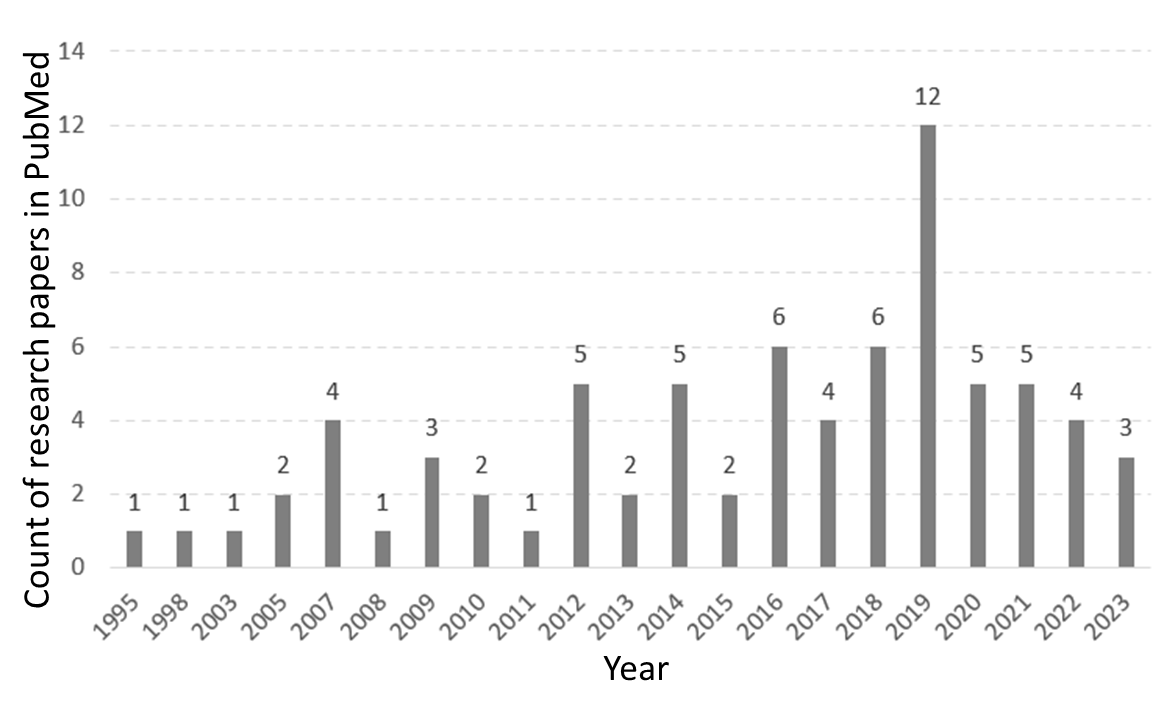
**

**S-Figure 1.** Counts of the research papers on VGSCs in cancers in PubMed by year.

**
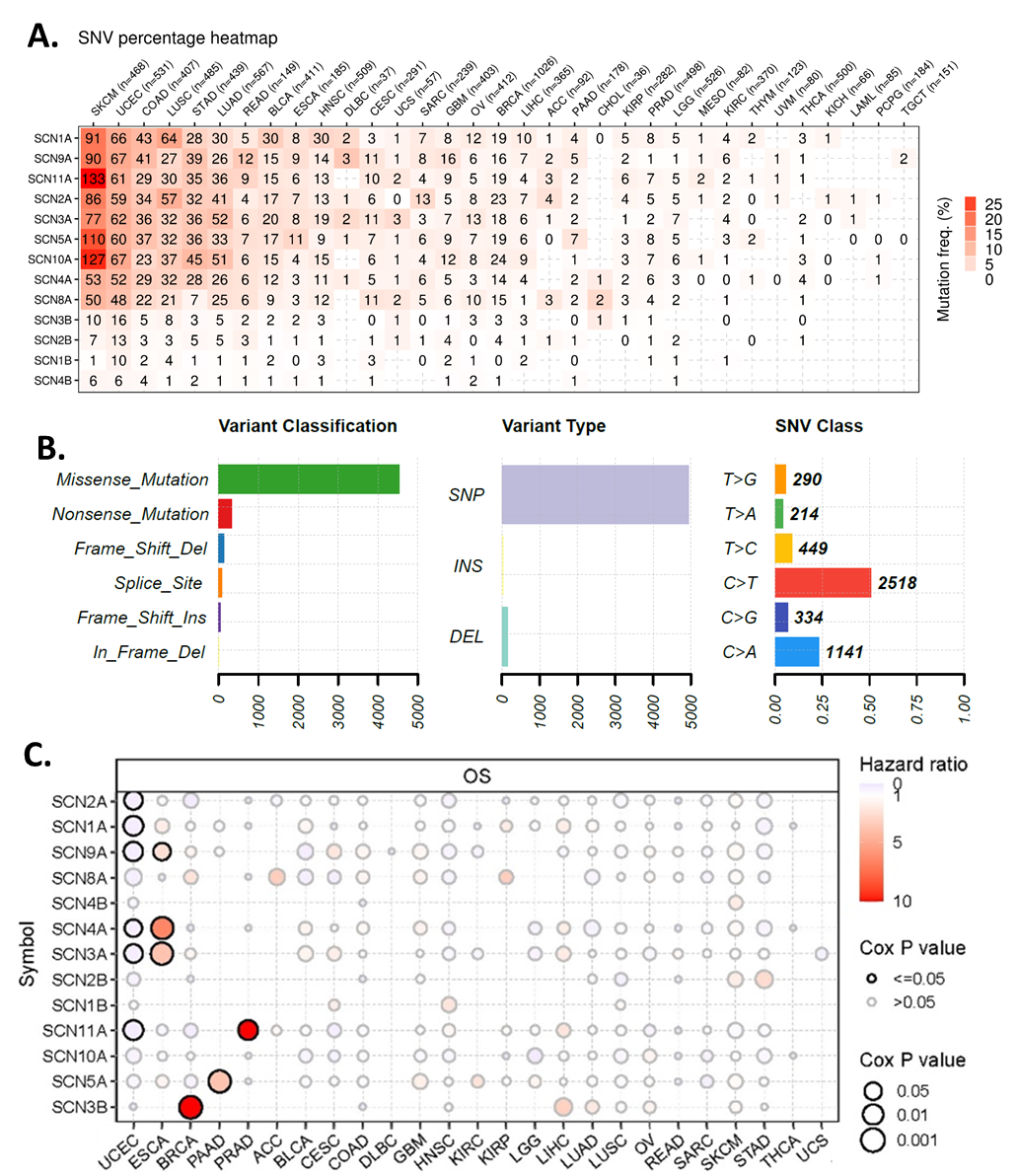
**

**S-Figure 2.** Single nucleotide variant (SNV) of VGSC in cancers. **A.** SNV percentage heatmap of VGSC in cancers. The heatmap summarizes the frequency of deleterious mutations, the number in the heatmap shows the exact number of mutation cases in cancer. The total cancer case number (n) was provided on top of the heatmap. The color represents the frequency calculated by the n/number of mutation cases. The genes and cancer types were displayed following the order of frequency. **B.** An overview of the SNV classes of the VGSC genes in cancers. **C.** Overall survival association of VGSC in cancers. The overall survival difference between mutant (deleterious) and wild type was compared.

**
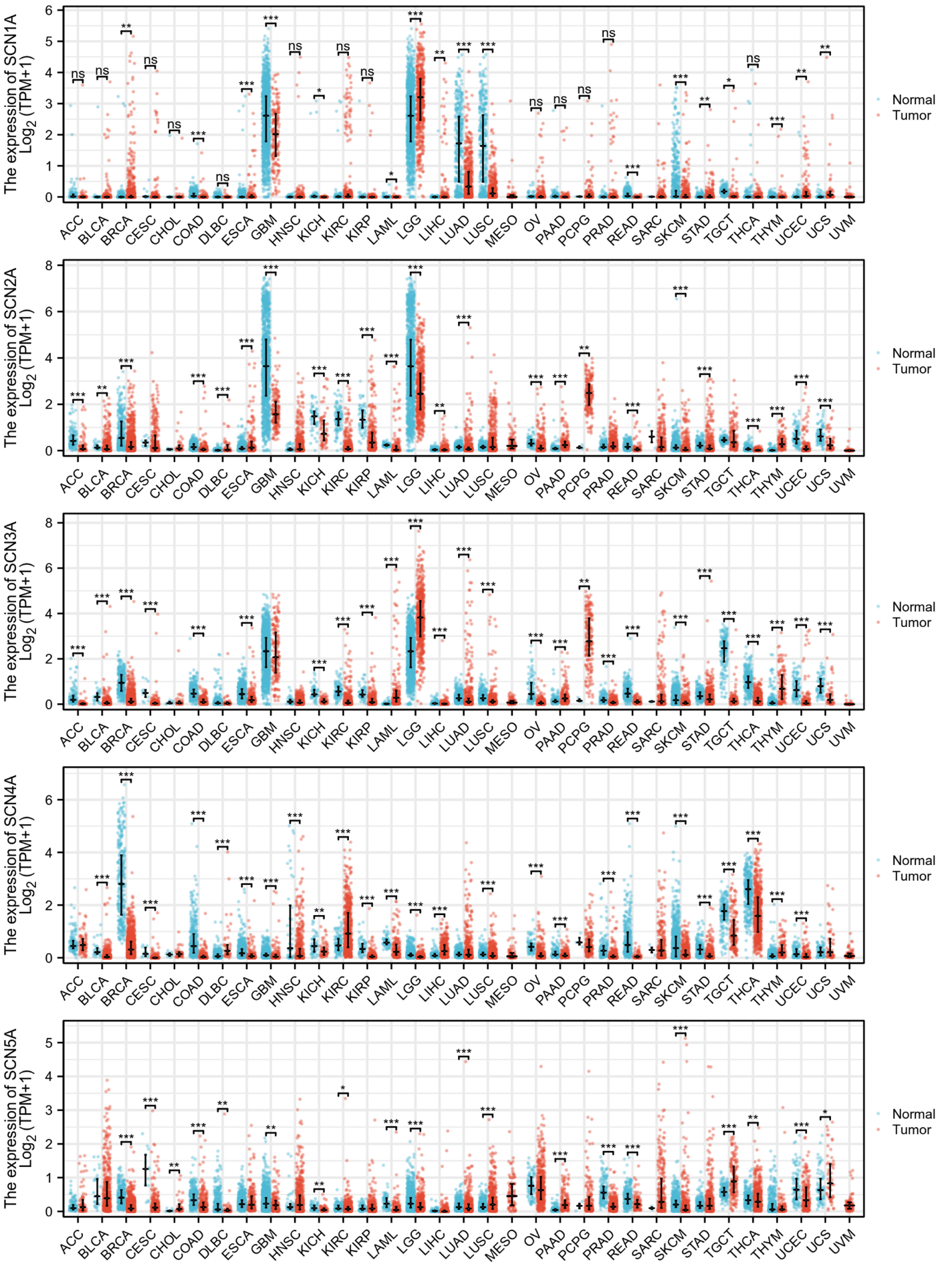
**

**
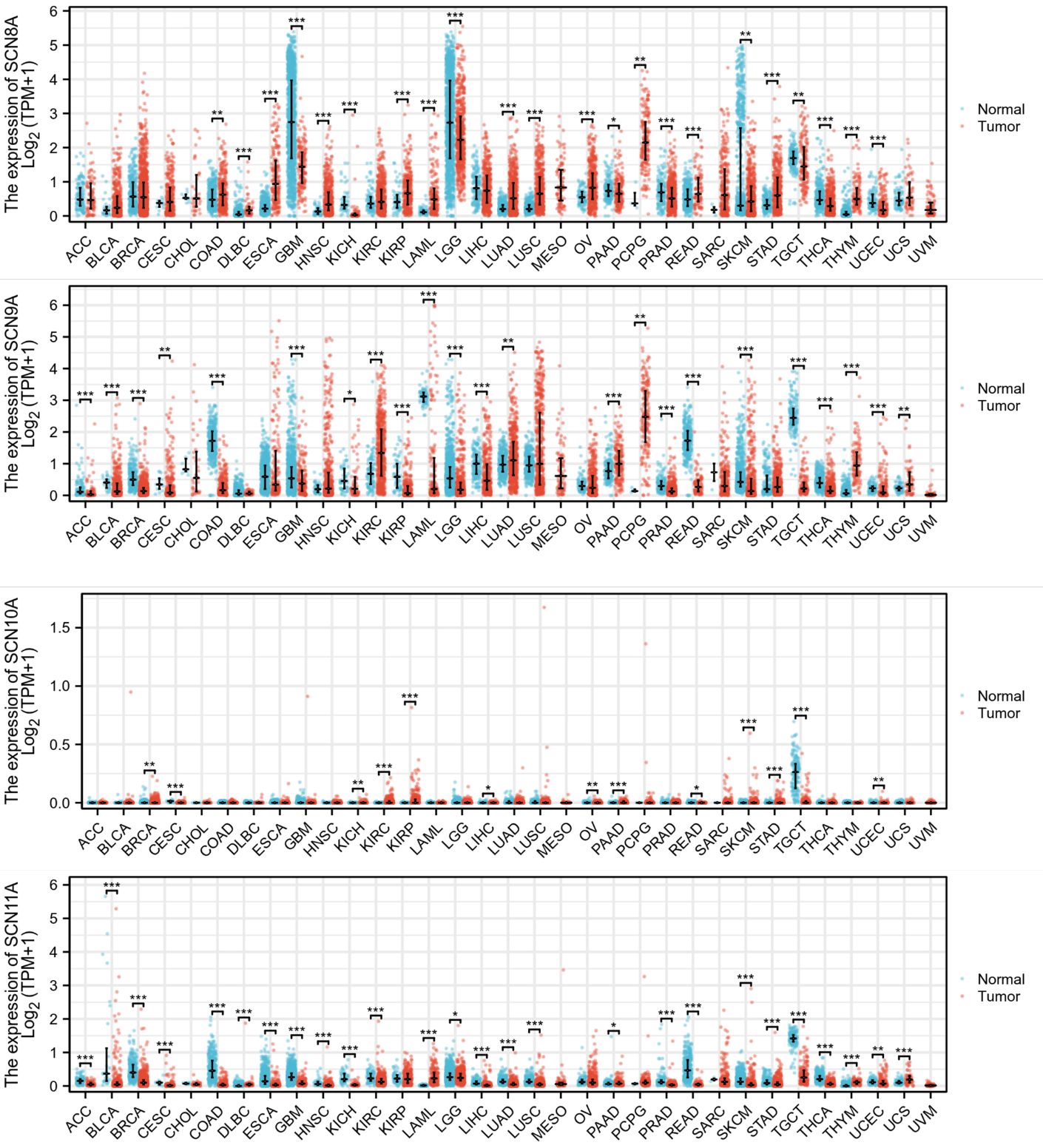
**

**
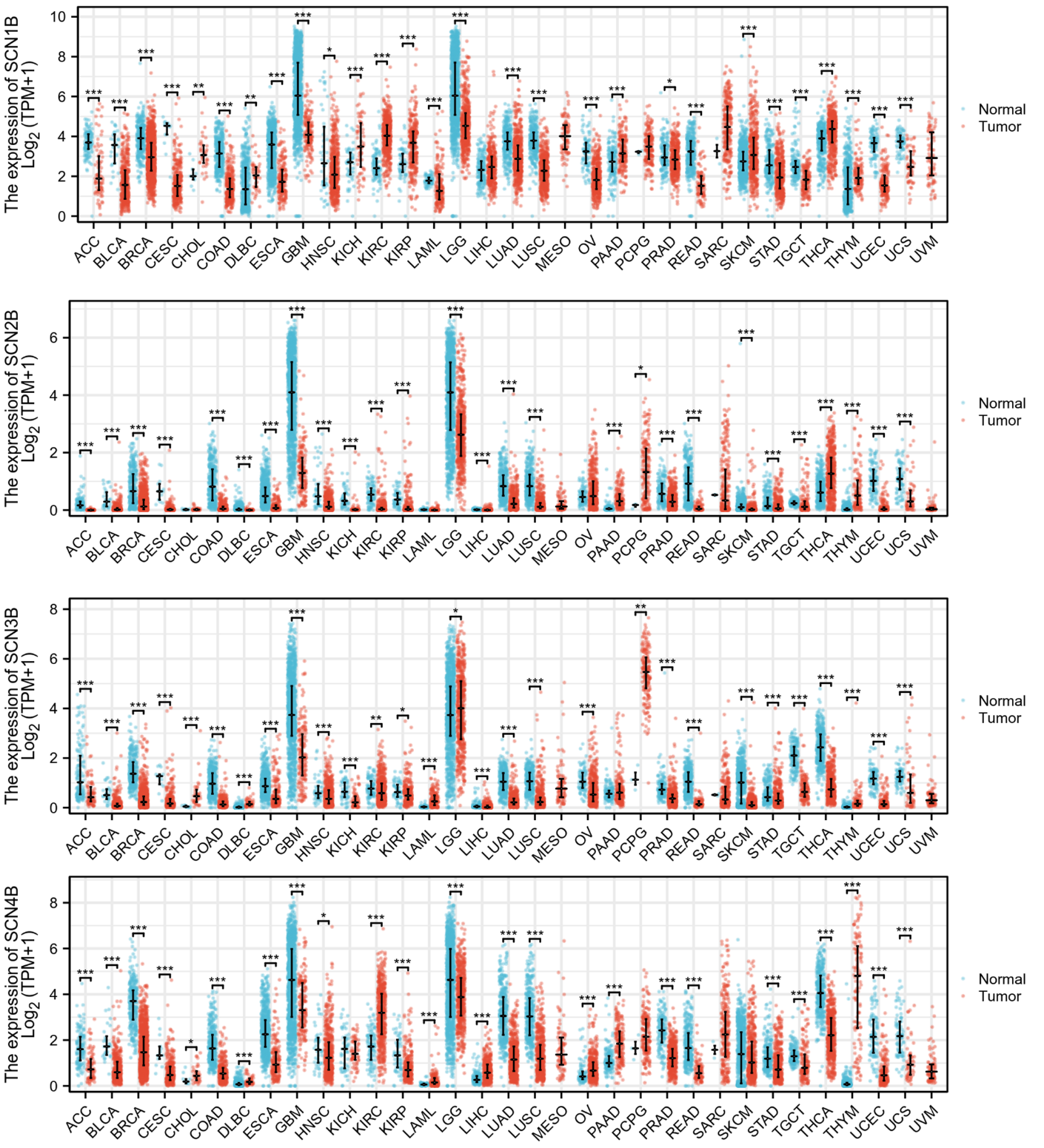
**

**S-Figure 3.** Expression of VGSC in tumour and normal tissues. TCGA and GTEx data were compared. T-test p-value *<0.05, **<0.01,***<0.001.


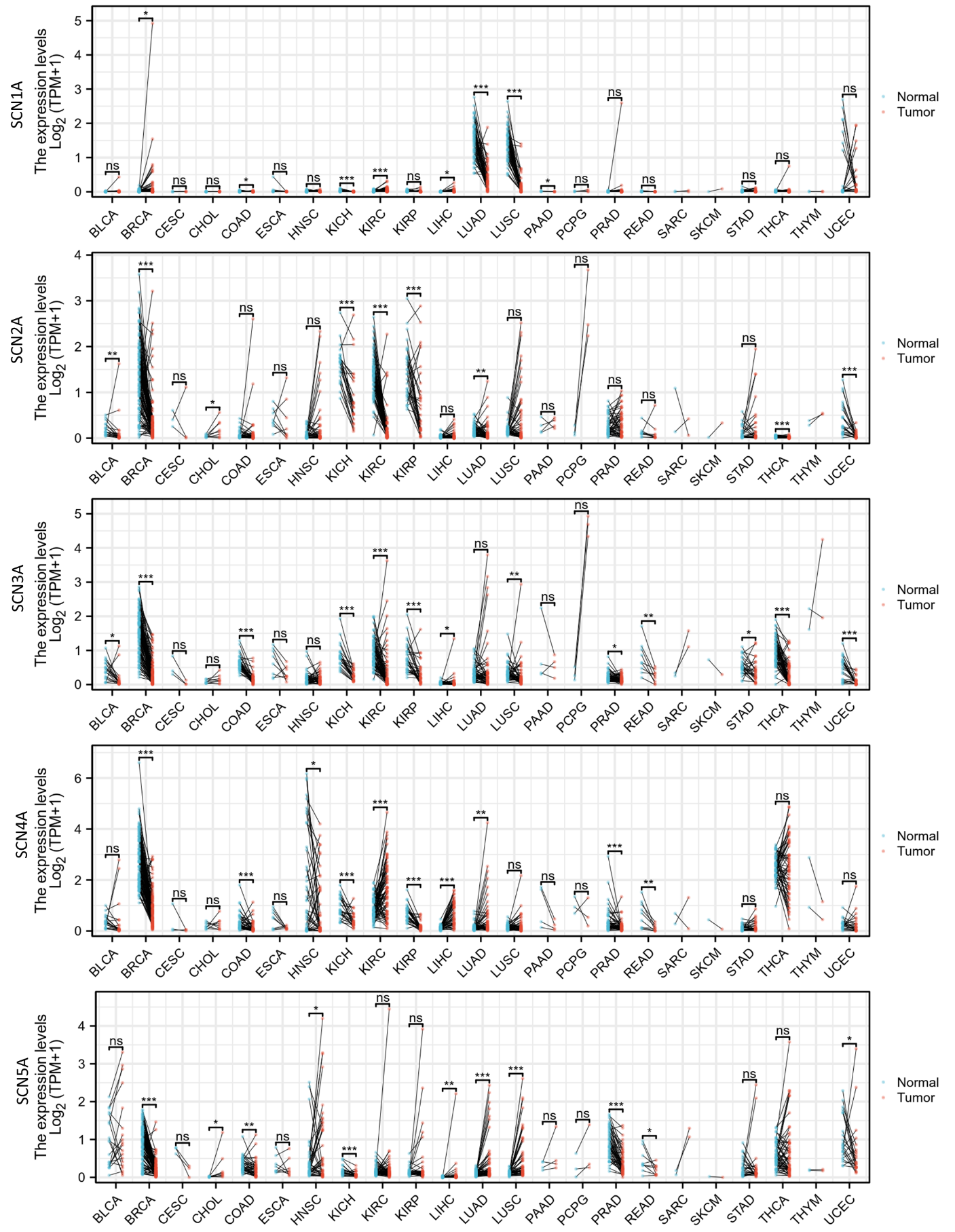


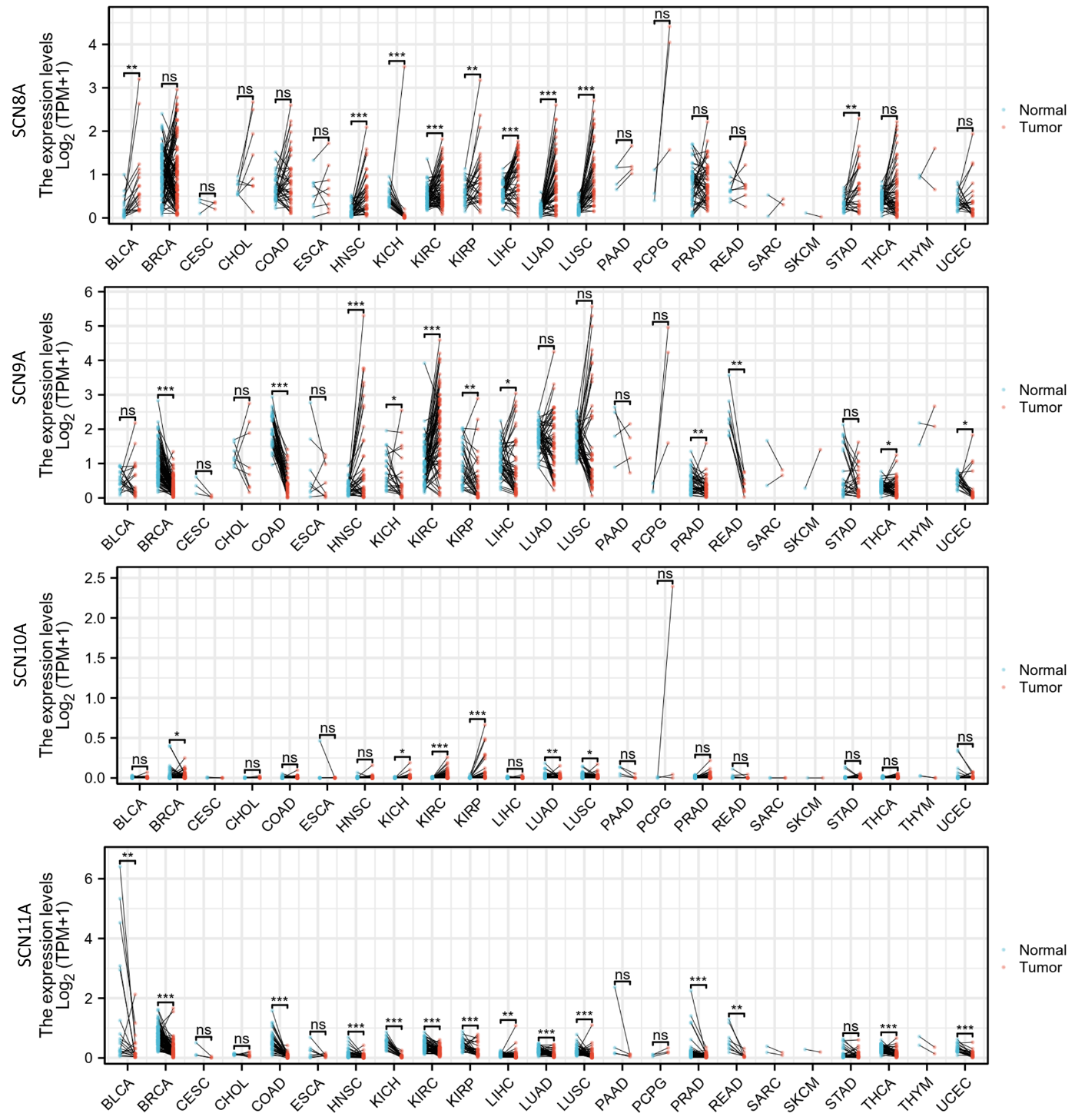


**
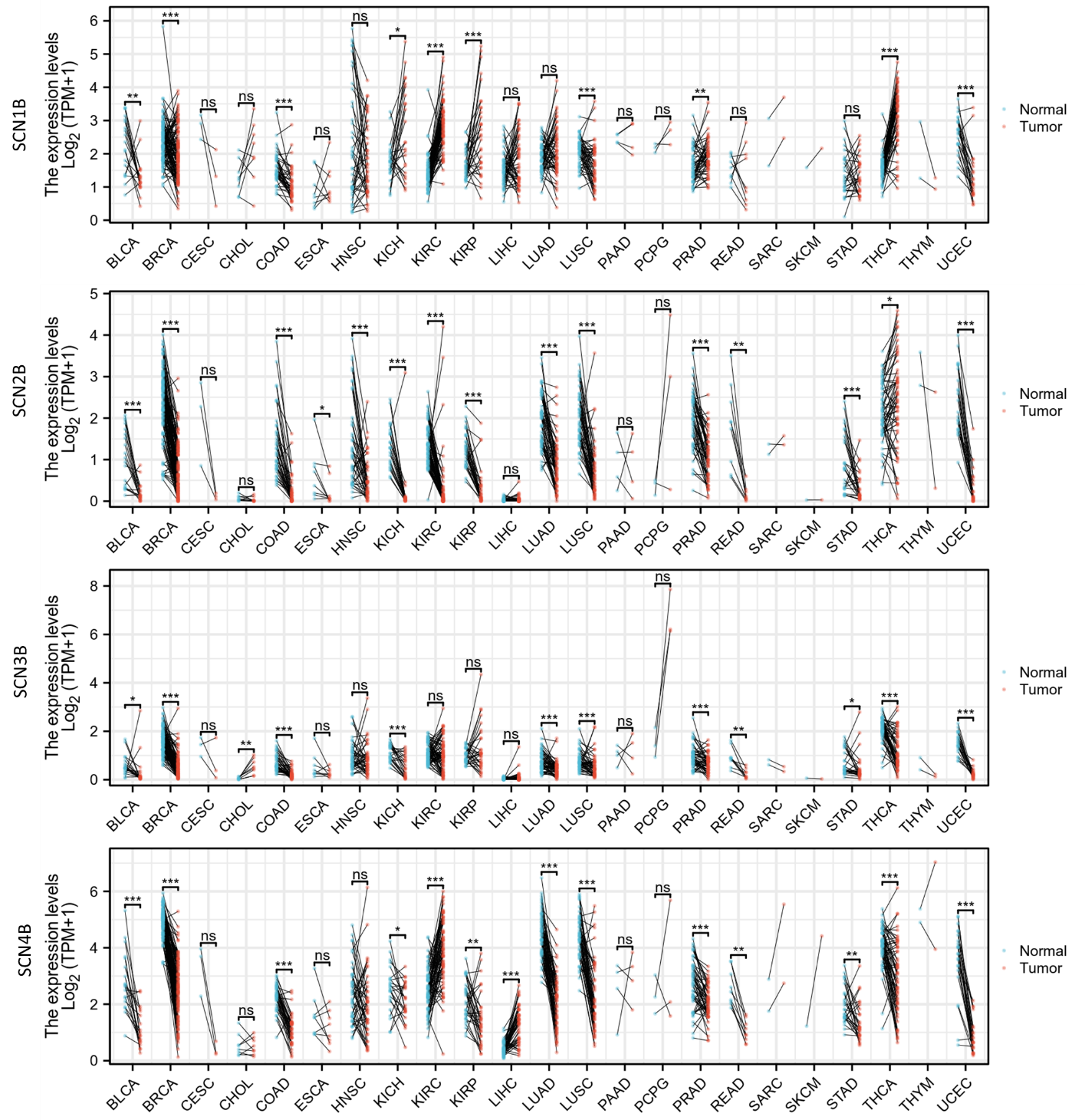
**

**S-Figure 4.** Expression of VGSC in tumour and normal tissues. TCGA and TCGA paired normal sample data were compared. Paired T-test p-value *<0.05, **<0.01,***<0.001. ns: not significant.

**
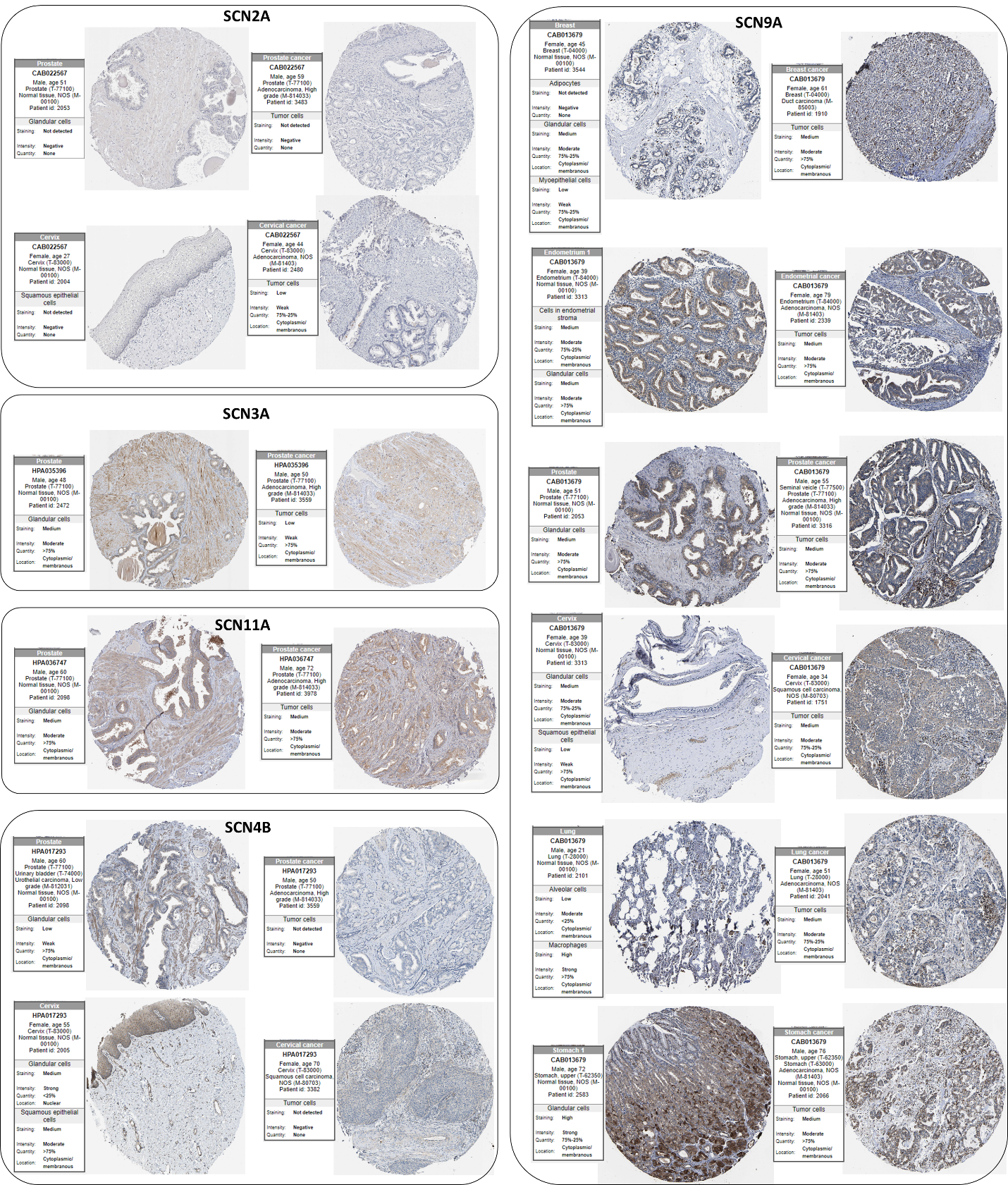
**

**S-Figure 5.** Protein expression of VGSC in tumor and normal tissues. Representative images of protein staining were shown. The Images were accessed from the Human Protein Atlas (HPA).

**
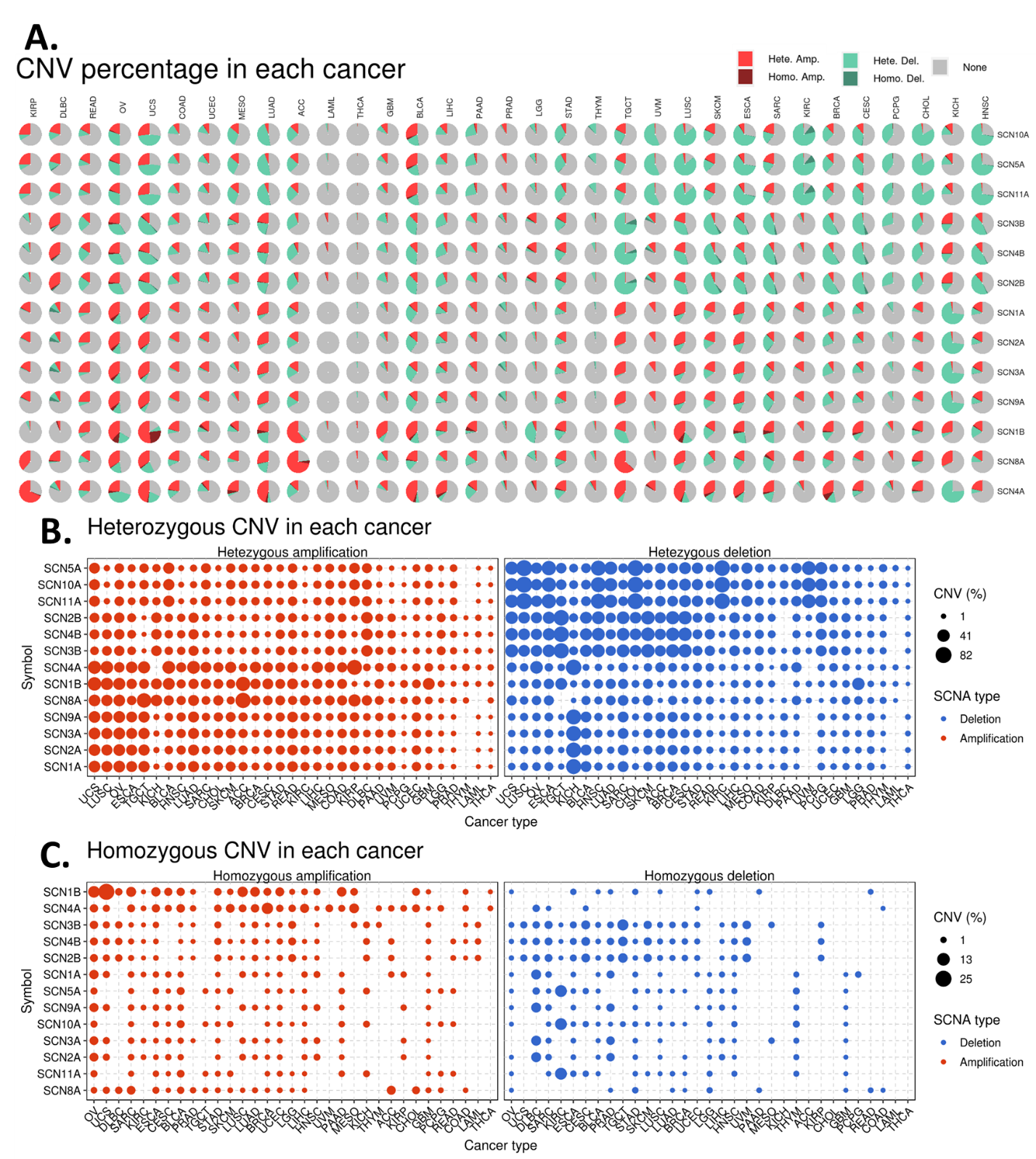
**

**S-Figure 6.** Copy number variant (CNV) profile of VGSC in cancers. **A.** CNV proportion pie plot showing the constitution of the Heterozygous (hete)/Homozygous (homo) CNV of VGSC in cancers. **B.** Bubble plot showing the percentage of heterozygous CNV, including heterozygous amplification and deletion of each gene in each cancer. **C.** Bubble plot showing the percentage of homozygous CNV, including heterozygous amplification and deletion of each gene in each cancer.

**
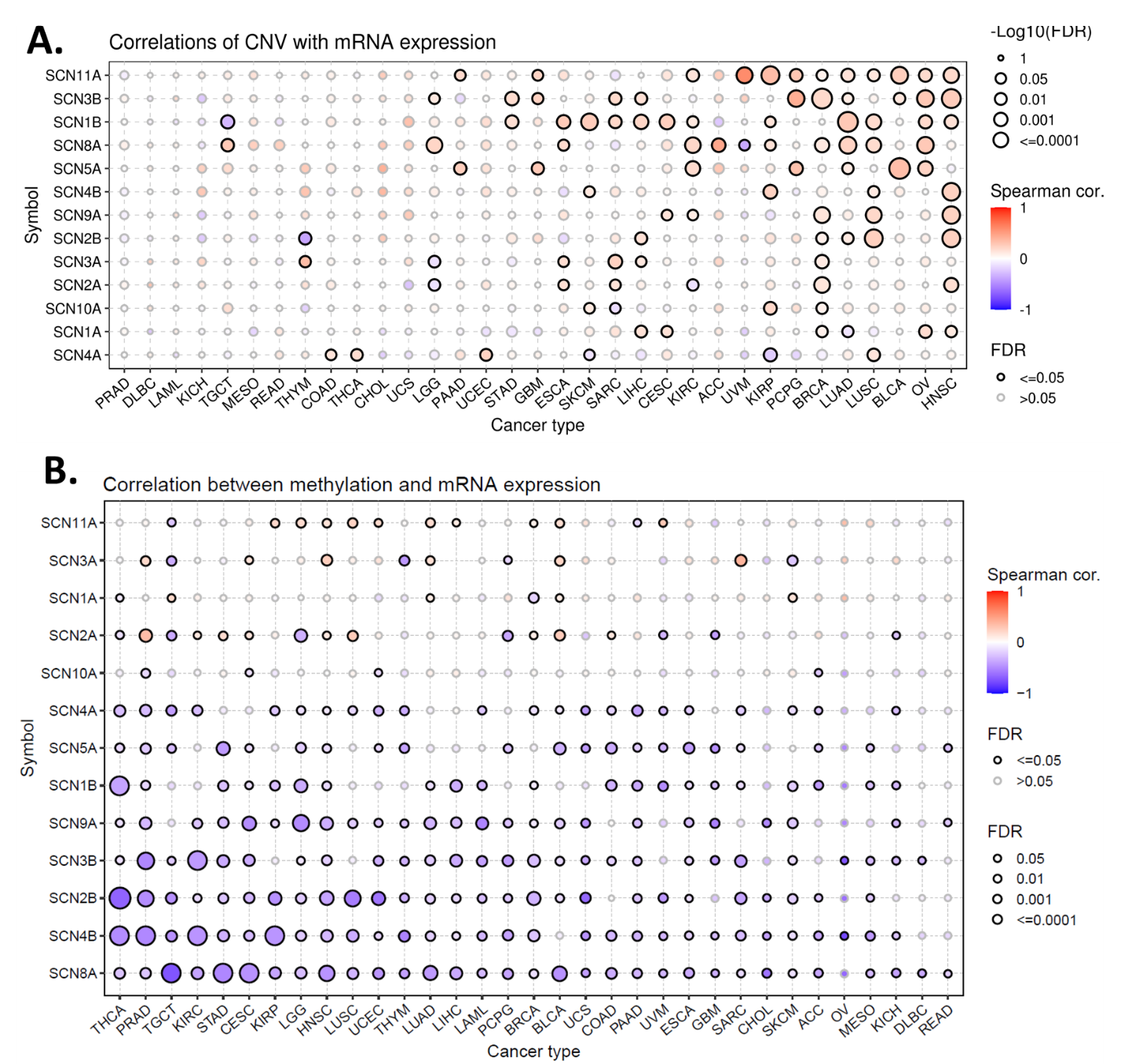
**

**S-Figure 7.** Potential expression regulation of VGSC by Copy Number Variation (CNV) and methylation in cancers. **A.** The correlations between CNV and mRNA expression of VGSC genes in cancers. Integrated CNV status was used in the calculation where “-2” represents a homozygous deletion, “-1” represents a heterozygous deletion, “0” represents diploid, “1” represents a heterozygous amplification, and “2” represents a homozygous amplification. **B.** The correlations between methylation and mRNA expression of VGSC genes in cancers. There are multiple methylation sites in the region of a gene and the most negatively correlated site were used to calculate the correlation. FDR: False Discovery Rate.

**
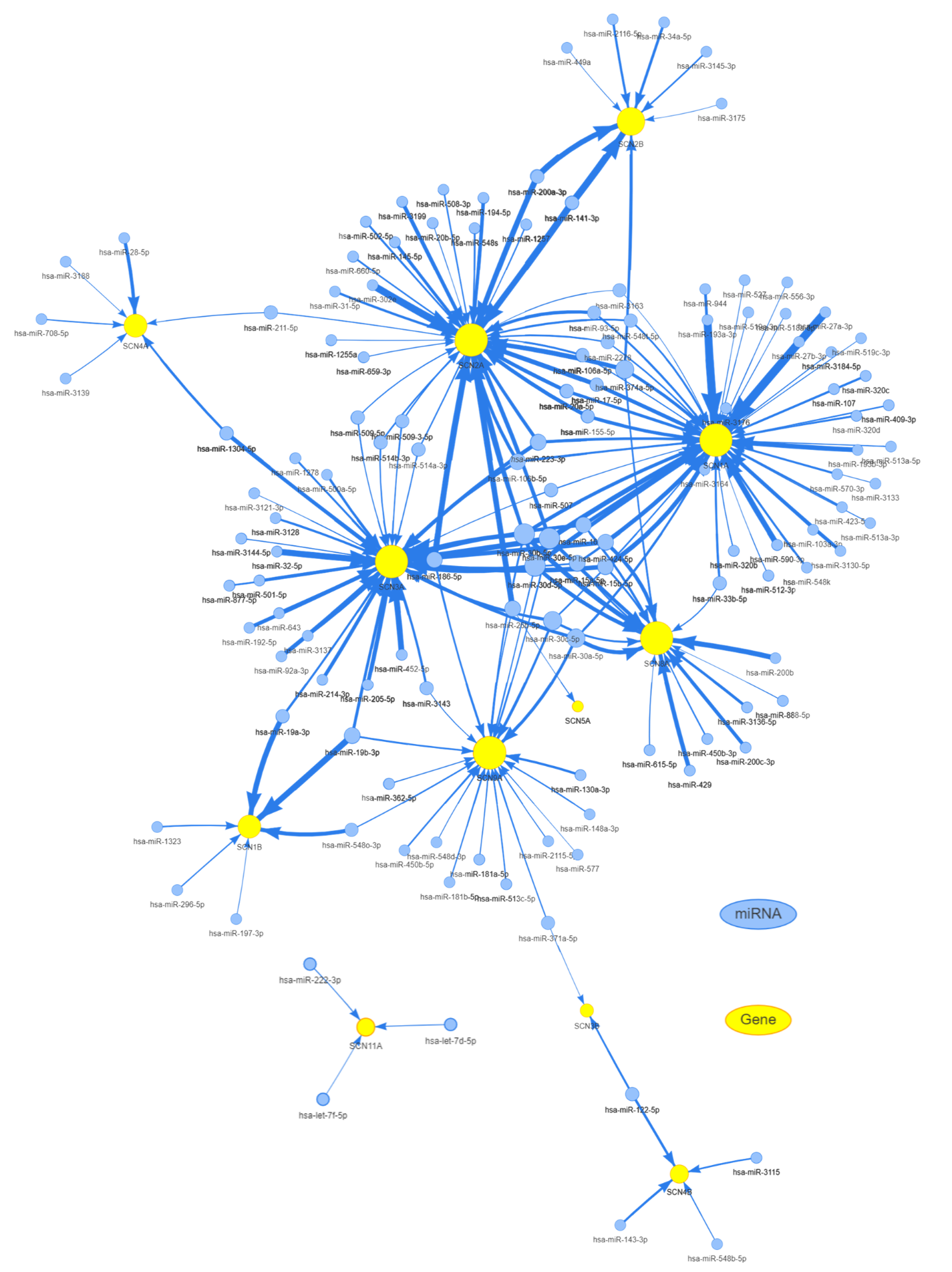
**

**S-Figure 8.** A miRNA regulation network of VGSC in cancers. The regulation relationship of miRNA-gene was collected from experimentally verified data (papers, TarBase, miRTarBase, mir2disease) and predicted data (targetscan and miRanda). miRNA transcript expression data were collected from TCGA. The expression of miRNA or mRNA was merged by TCGA barcode and Person's correlation between paired mRNA and miRNA expression was calculated. P-value was adjusted by FDR and genes with FDR<=0.05 and R<0 were plotted in the network using R packages visNetwork. Nodes in this network represent miRNAs and genes, with edges signifying the regulation of miRNA to gene. Node size is directly proportional to the node's degree, while edge width is determined by the absolute value of the correlation coefficient between miRNA and mRNA expression.

**
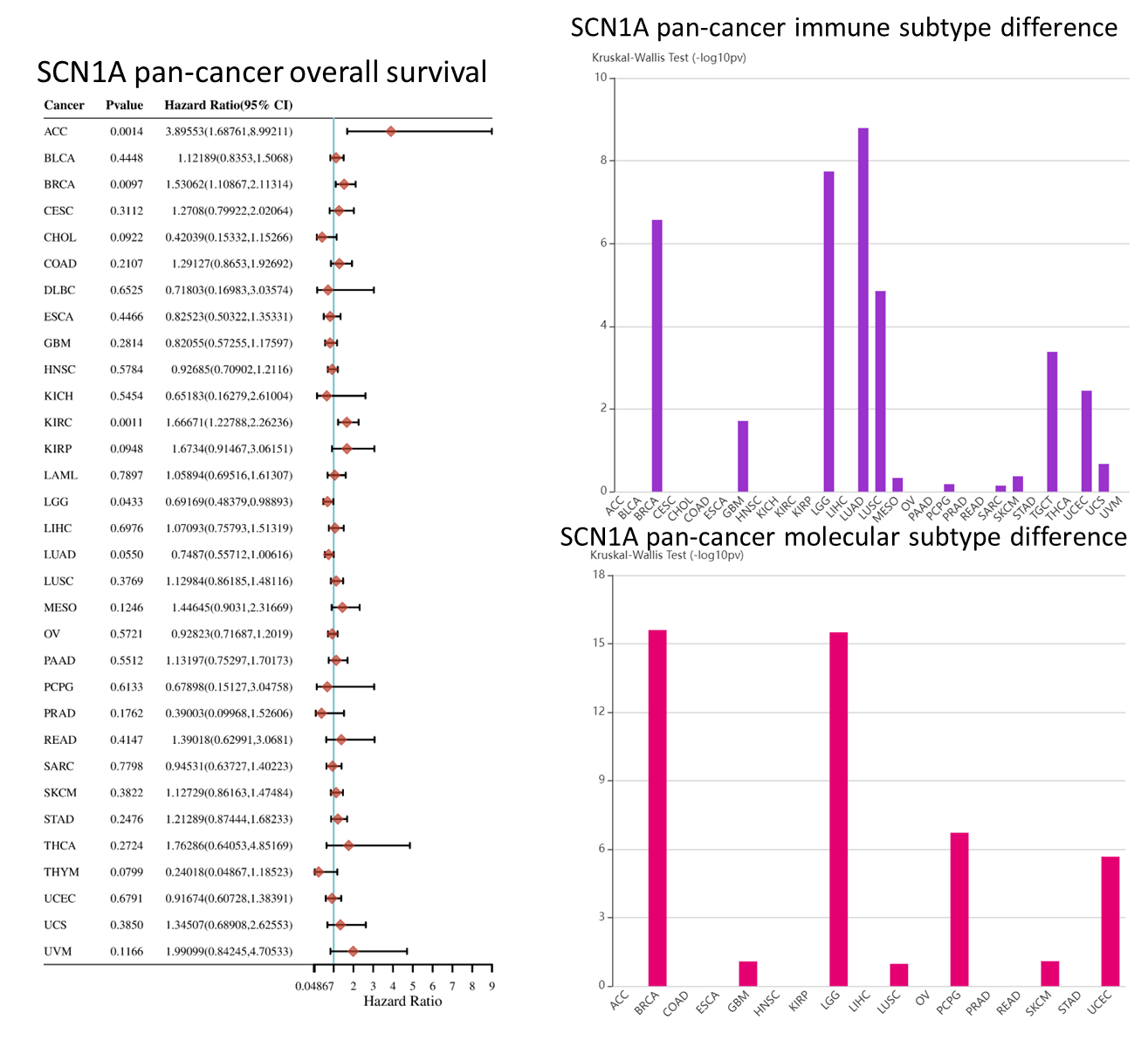
**

**
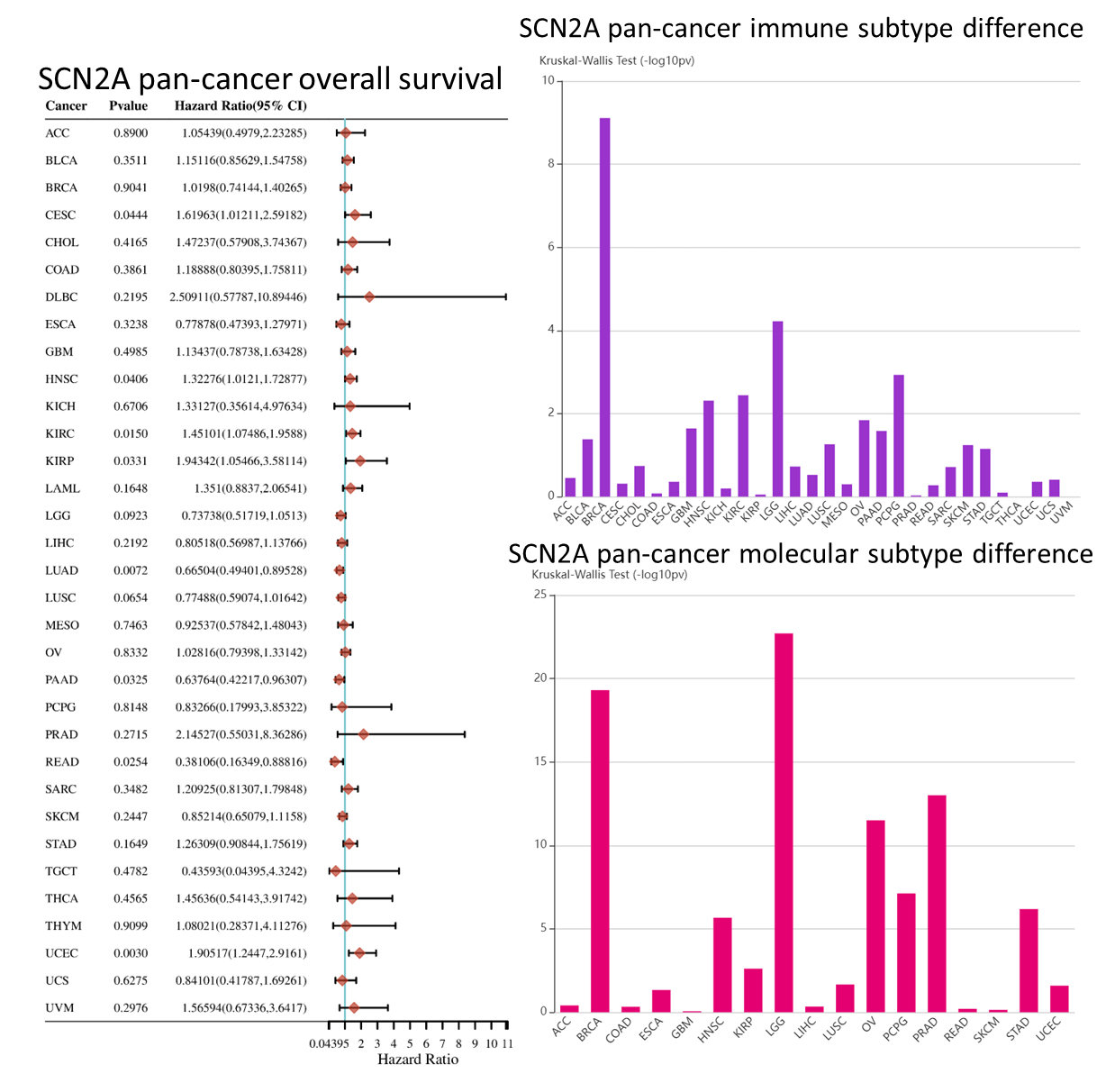
**

**
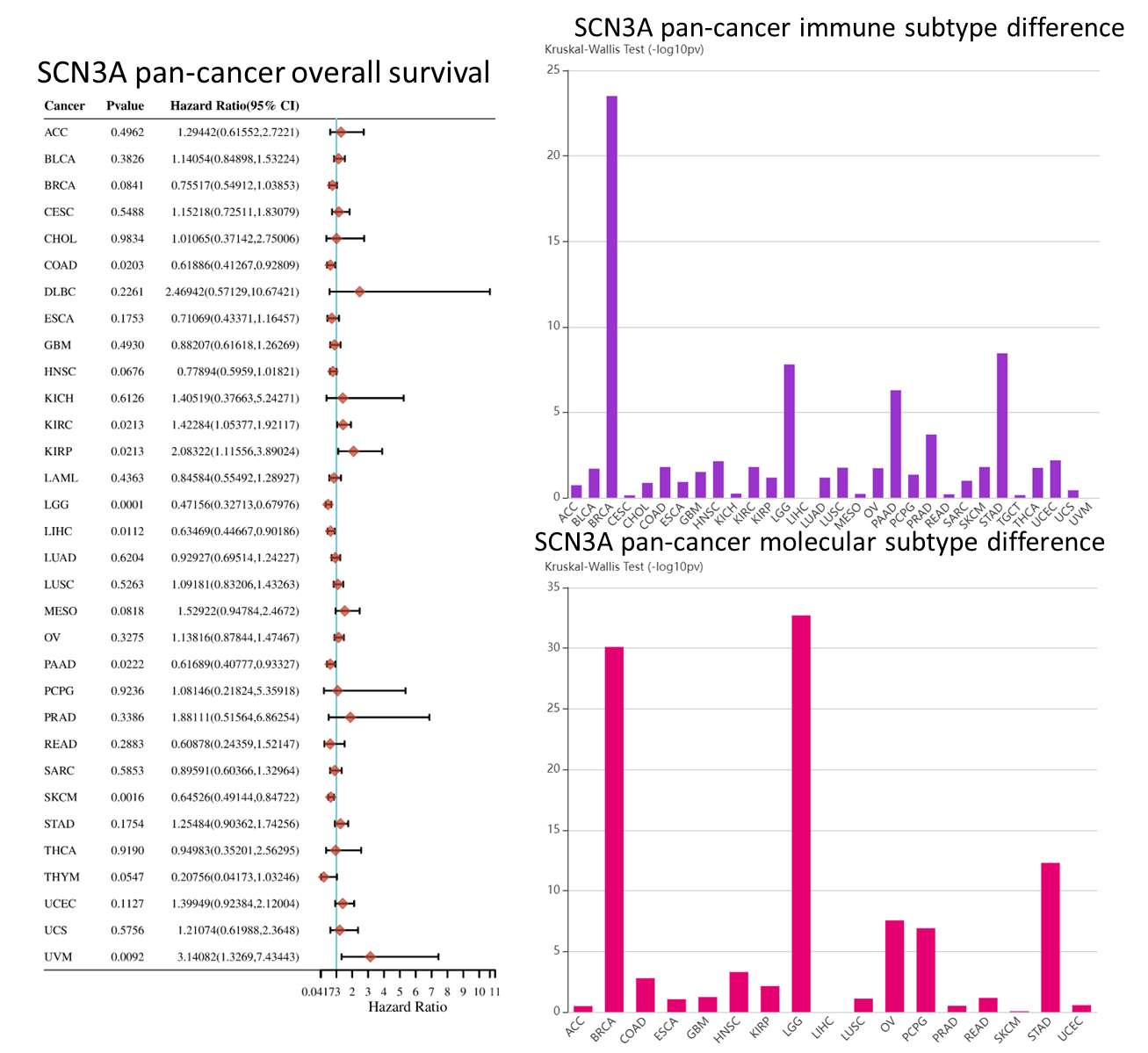
**

**
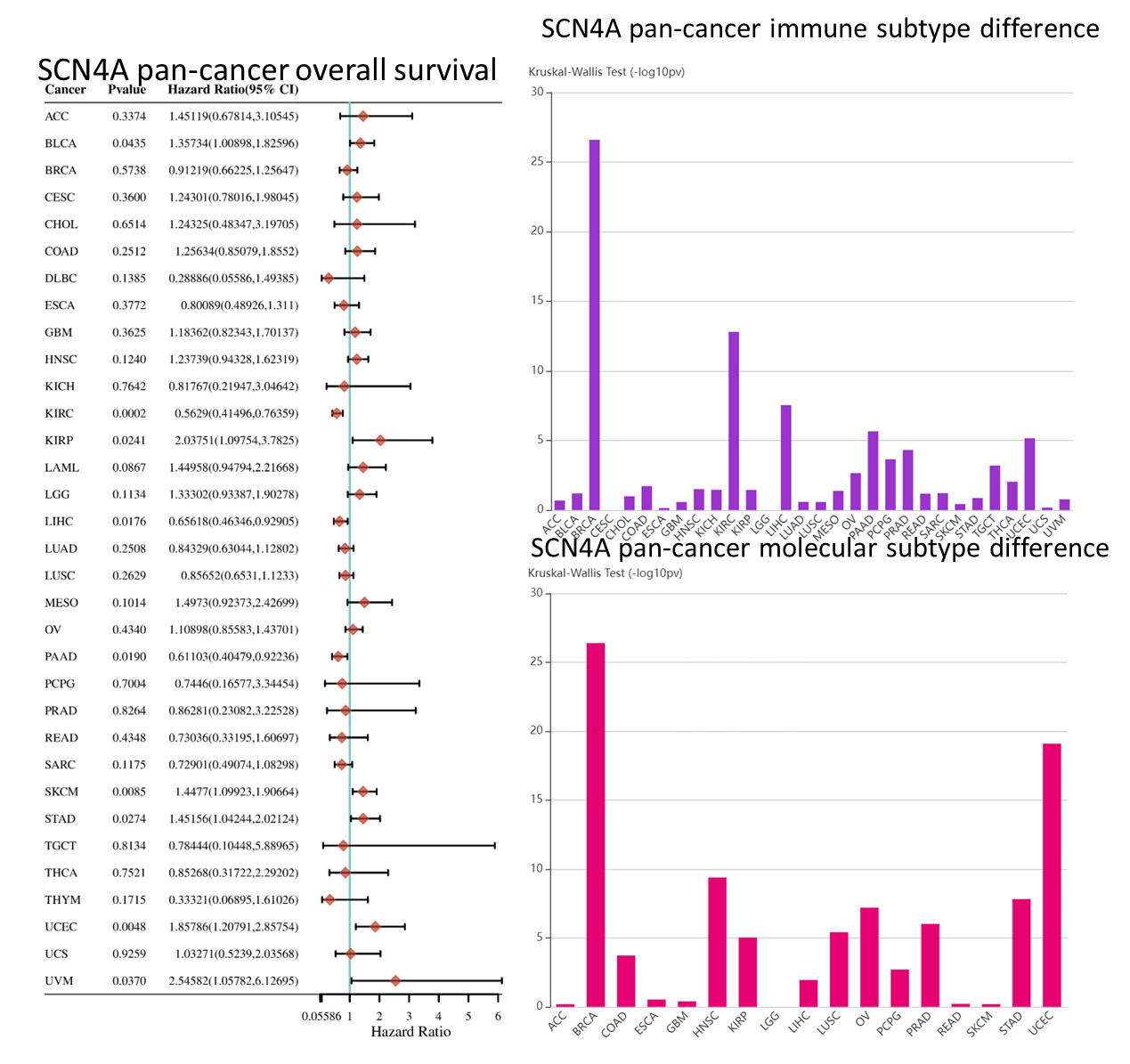
**

**
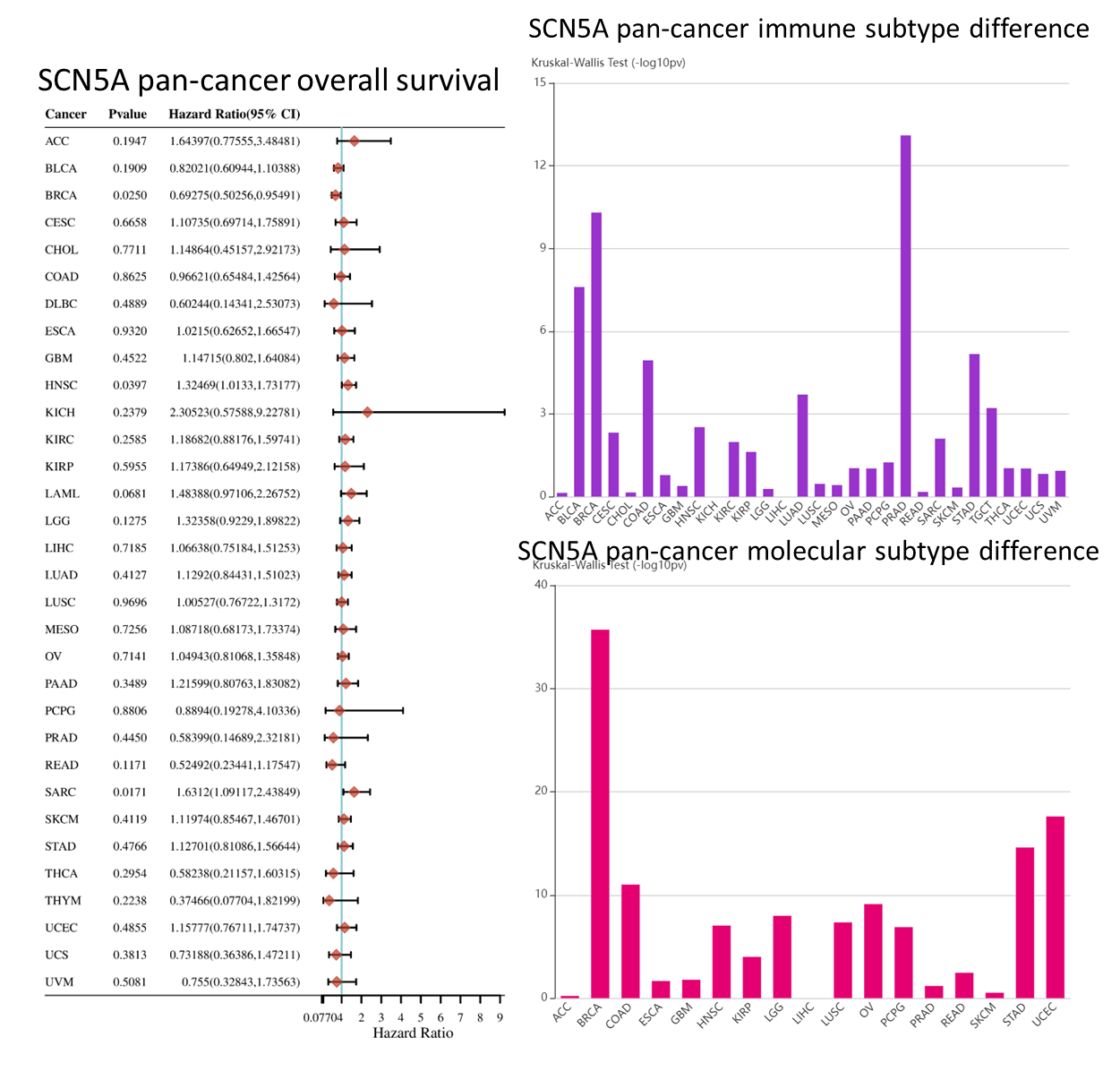
**

**
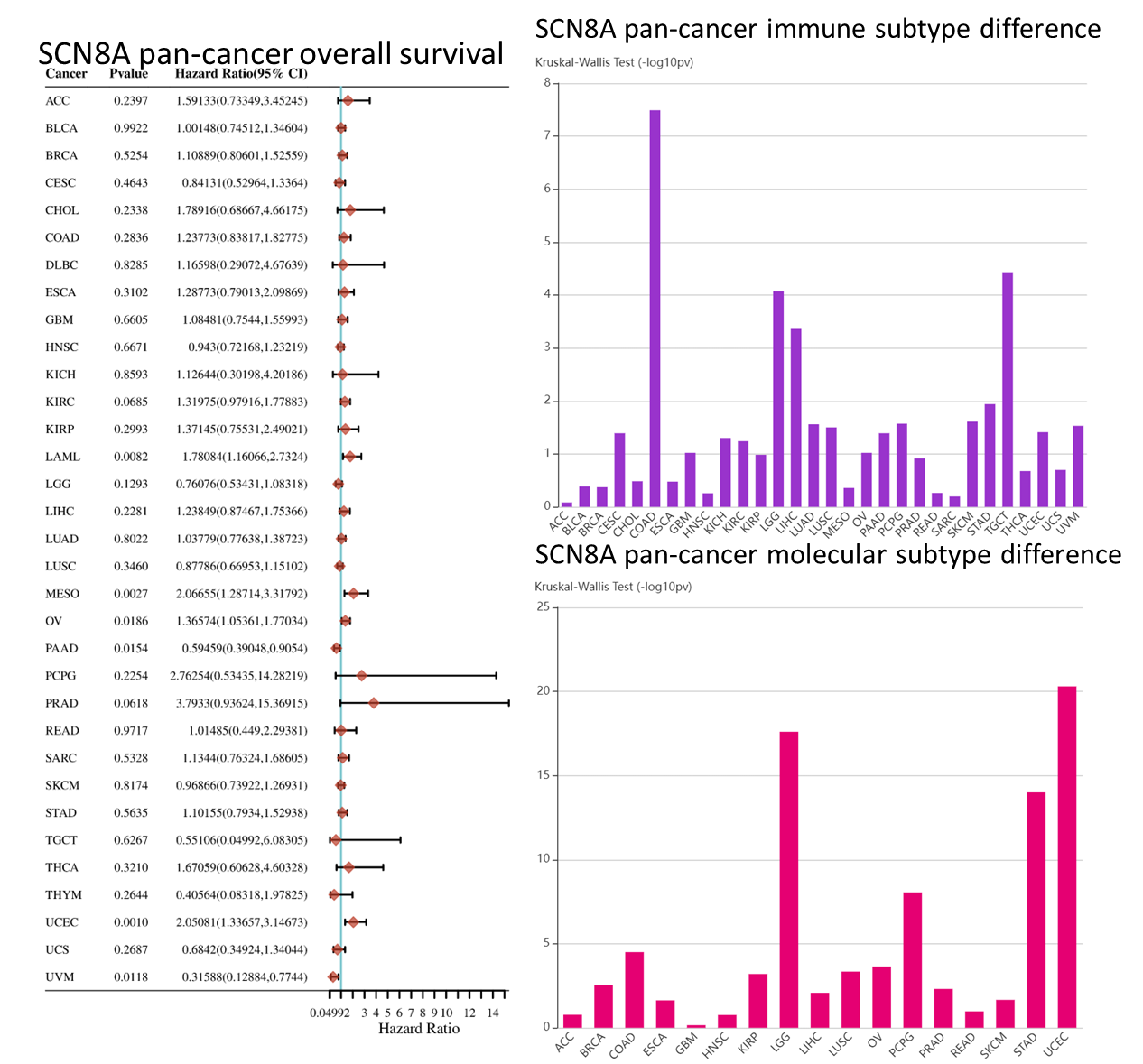
**

**
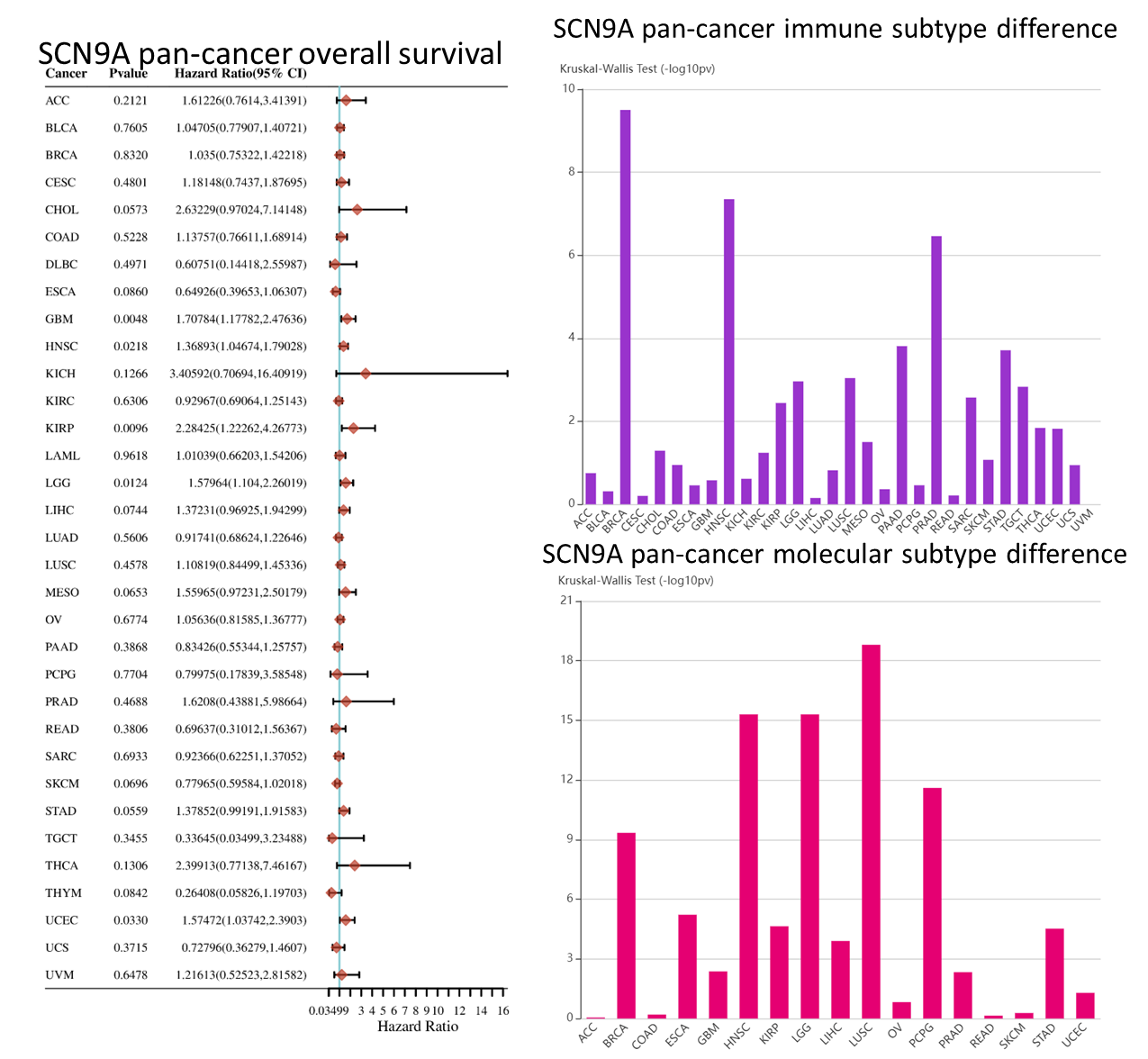
**

**
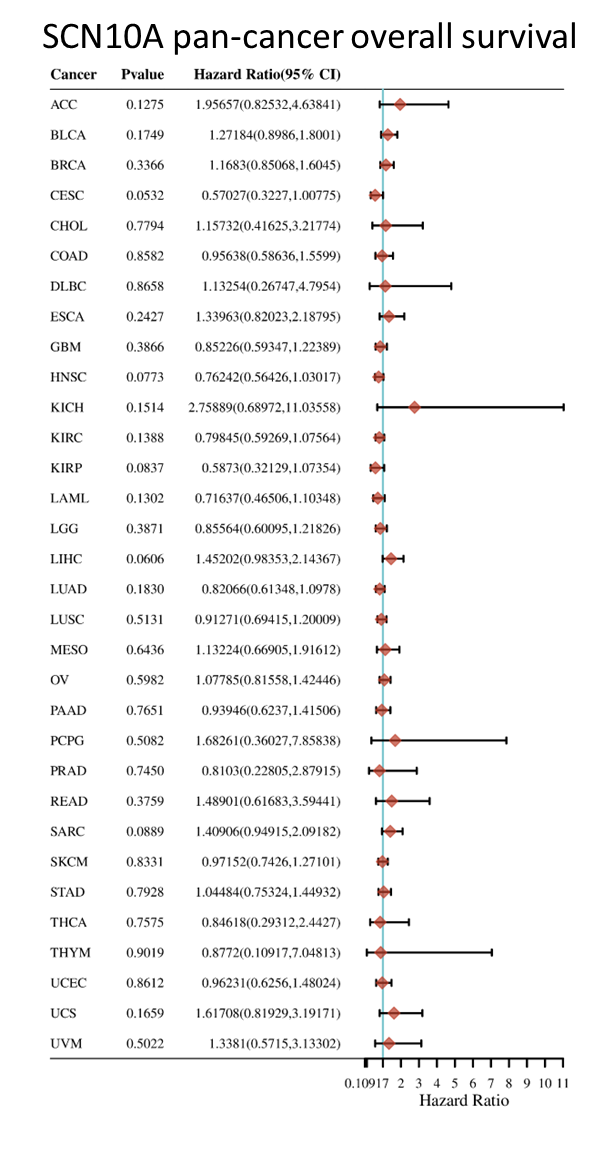
**

**
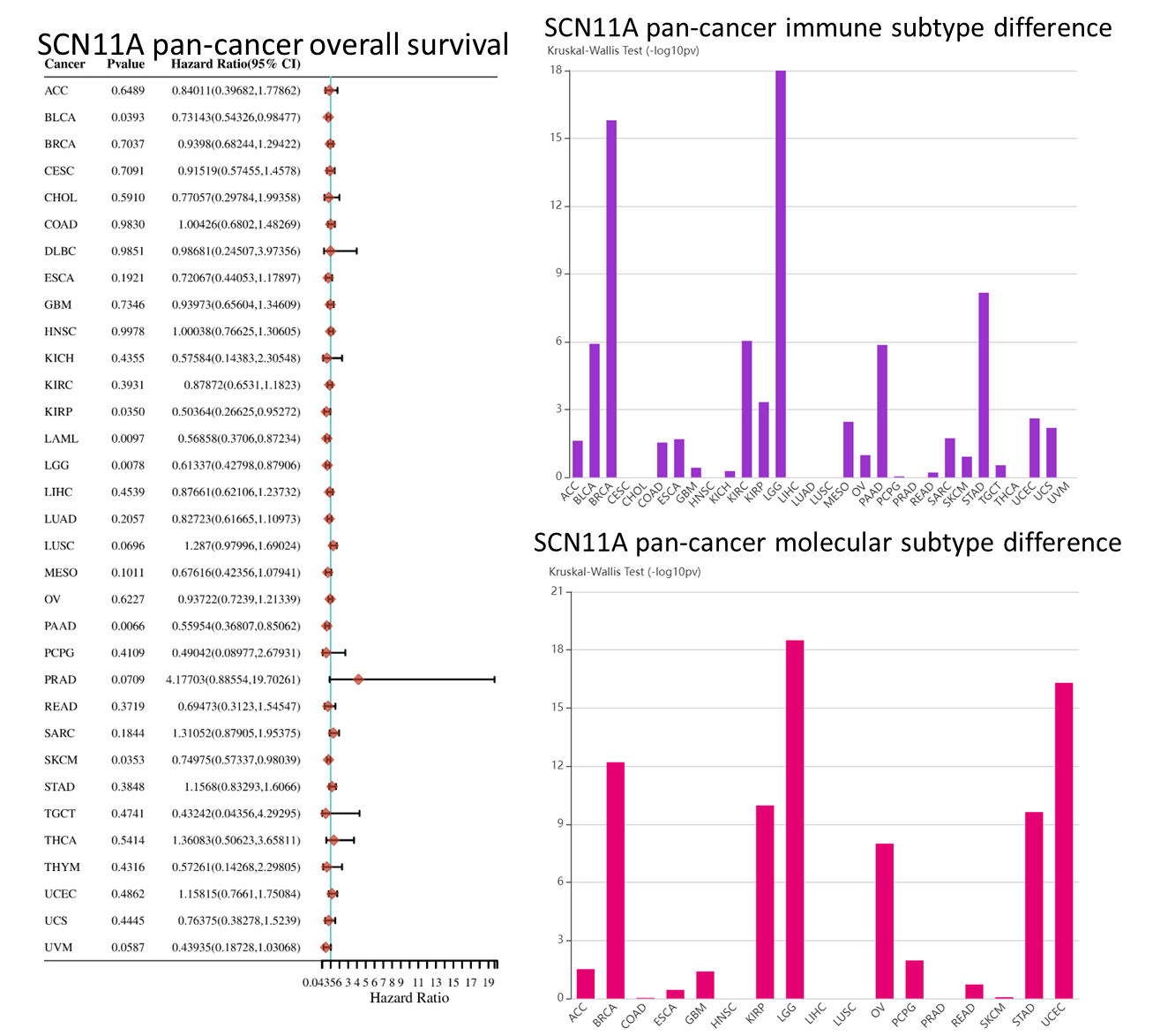
**

**
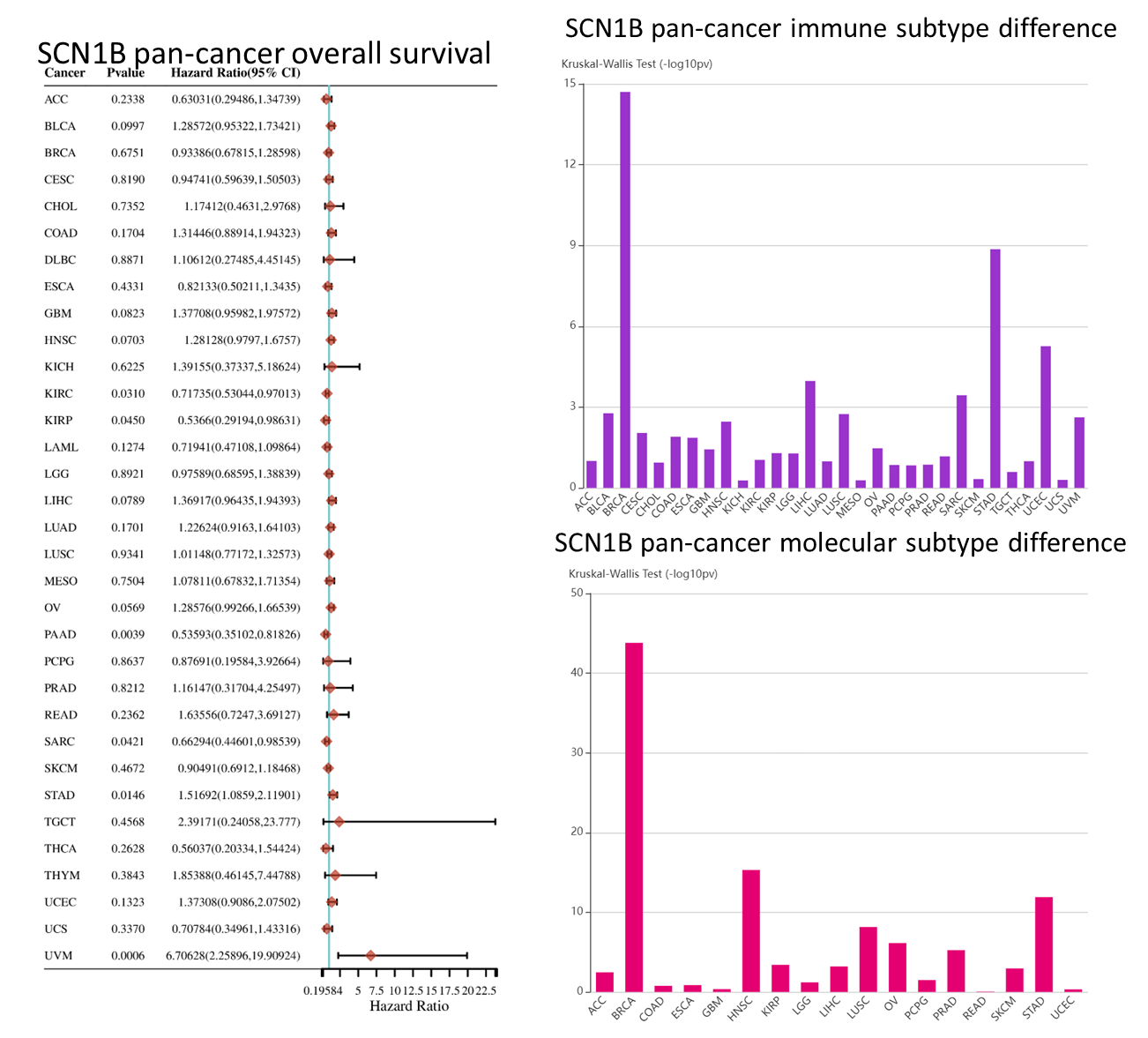
**

**
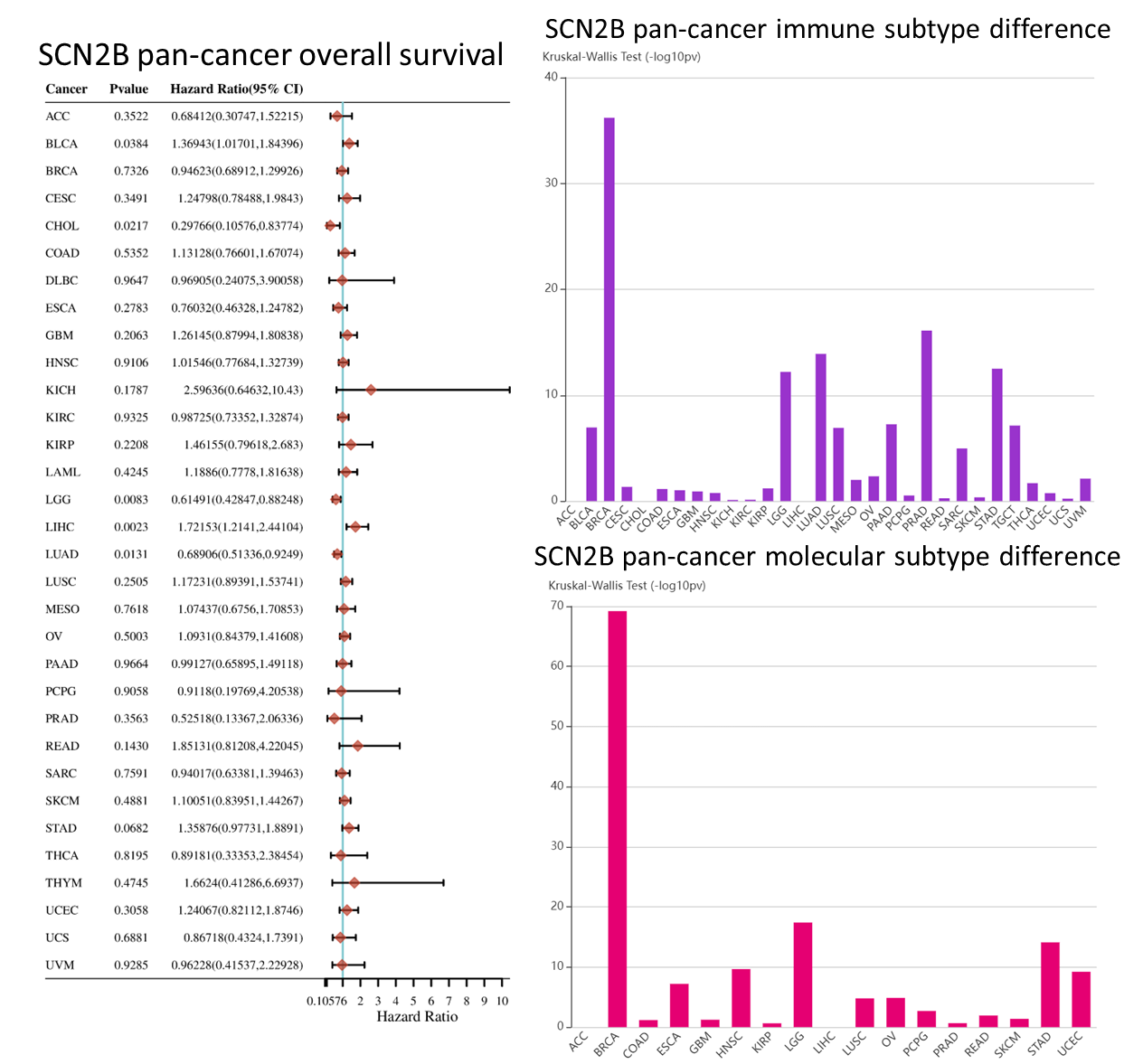
**

**
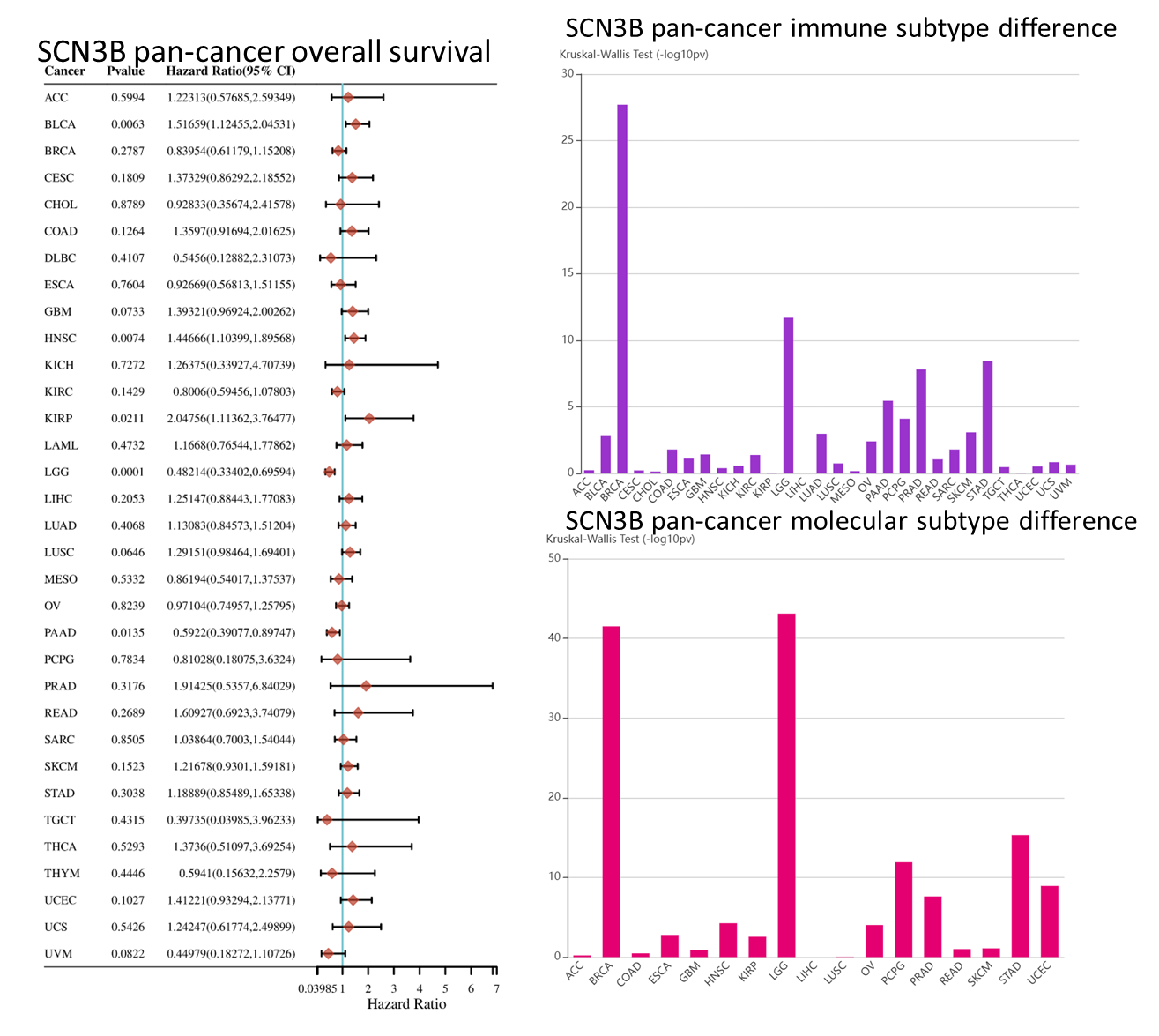
**

**
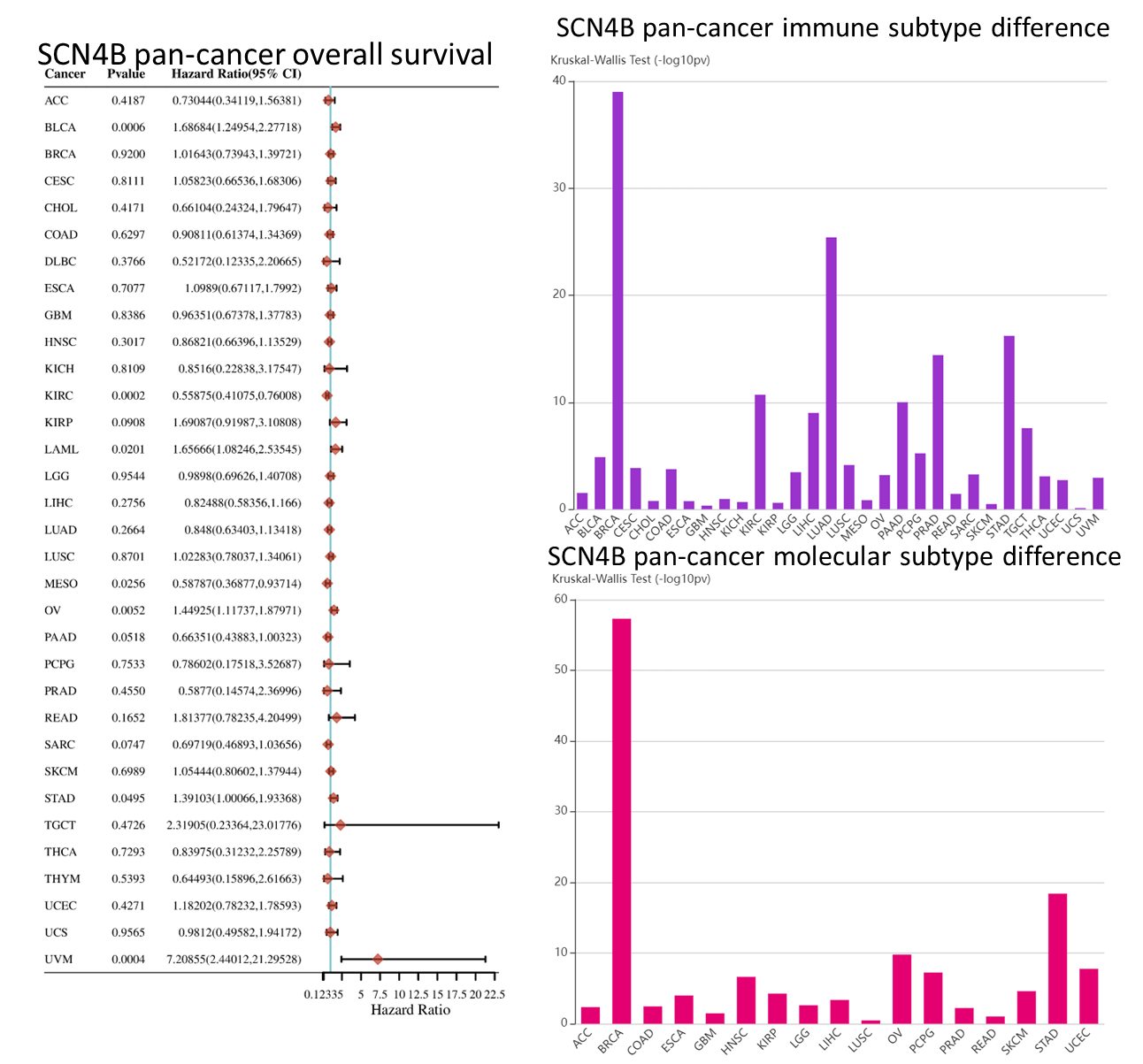
**

**S-Figure 9.** Clinical association of VGSC in cancers. Survival association, immune subtype association, and molecular subtype association of VGSC in cancers were analyzed. The subtype association of SCN10A was not available in the database. For each subfigure: **Left panel:** Survival association of VGSC in cancers. The survival of patients of high and low expression (separated by median) in the cancer was compared. **Right top panel:** immune subtype association, the p-values were plotted. **Right below panel:** molecular subtype association, the p-values were plotted. The immune subtype association and molecular subtype association of VGSC in cancers were analyzed using the TISIDB web portal.


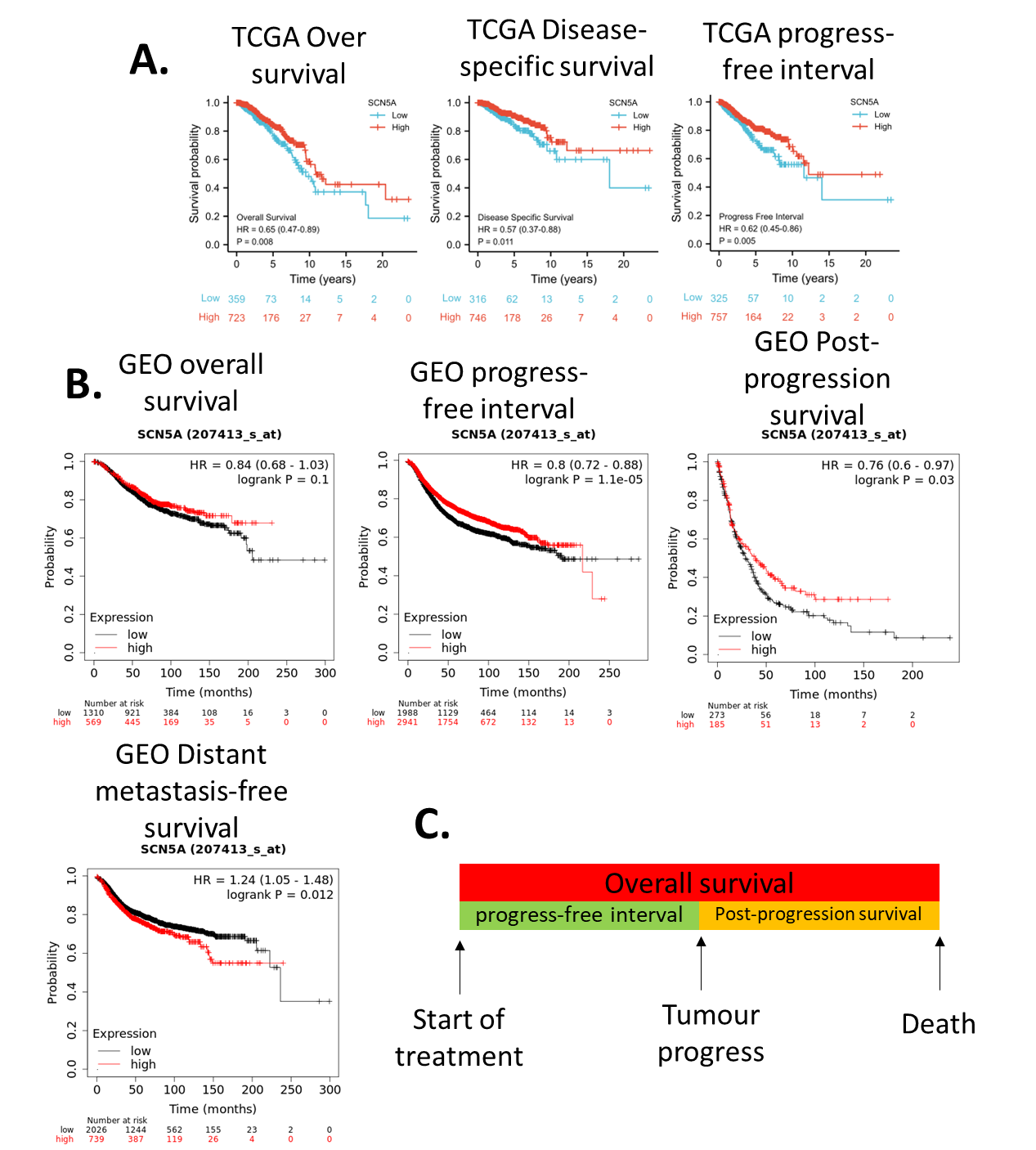


**S-Figure 10.** Survival association of SCN5A in breast cancer. **A.** survival data of TCGA. **B.** Survival data of GEO data integrated by Kaplan-Meier Plotter. **C.** Relationship of overall survival, progress-free interval, and post-progression survival.
